# Biomimetic Coacervate Coatings: From Phase Separation Fundamentals to Advanced Biomedical Applications

**DOI:** 10.3390/biomimetics11090647

**Published:** 2026-09-09

**Authors:** Ki Ha Min, Yi-Rang Jeong, Jong Won Mun, Kyu Ho Jeon, Seung Pil Pack

**Affiliations:** 1 Department of Biotechnology and Bioinformatics, Korea University, Sejong 30019, Republic of Korea; alsrlgk@gmail.com (K.H.M.);; 2 Institute of Industrial Technology, Korea University, Sejong 30019, Republic of Korea

**Keywords:** coacervate, surface coating, biomimetic, phase separation, biomedical applications

## Abstract

This review systematically elucidates the rapidly evolving field of biomimetic coacervate coatings, bridging the fundamental thermodynamic principles of liquid–liquid phase separation (LLPS) with advanced biomedical translations. While conventional surface modifications for medical implants frequently fail to maintain structural and functional integrity within dynamic, wet physiological environments, biomimetic coacervation inspired by natural underwater adhesive mechanisms offers a highly versatile, conformable, and robust interfacial strategy. Here, we analyze the critical physicochemical driving forces governing coacervate formation, emphasizing the synergistic interplay of electrostatic, hydrophobic, hydrogen-bonding, and cation–π interactions. We comprehensively discuss diverse macromolecular design principles utilizing marine-derived biopolymers, synthetic or recombinant polypeptides, and hybrid organic–inorganic condensates, alongside key architectural orchestration methodologies including direct deposition, in situ triggerable coacervation, and layer-by-layer (LbL) assembly. Furthermore, we evaluate multi-functional clinical translations, highlighting breakthroughs in wet tissue sealing, bone repair, localized stimuli-responsive drug or nucleic acid delivery, anti-biofouling medical device coatings, and regenerative cell–material interfaces. Ultimately, this review underscores the profound potential of biomimetic coacervates as a cornerstone platform for next-generation multifunctional medical devices and personalized regenerative medicine.

## 1. Introduction

The rapid advancement of modern medicine has driven the development of sophisticated biomedical devices, tissue engineering scaffolds, and implantable systems [1,2,3,4]. The clinical success of these devices relies heavily on their surface properties, as the interface between the synthetic material and the host biological environment dictates the subsequent cellular responses, immune compatibility, and long-term integration [1,5,6,7]. To achieve optimal biointerface performance, various physical and chemical surface modification techniques including plasma treatment, chemical vapor deposition, and organic solvent-based polymer coating have been extensively utilized [8,9,10]. However, conventional coating strategies frequently suffer from critical limitations, such as non-uniform deposition on complex three-dimensional porous geometries, harsh processing conditions that may compromise the integrity of delicate substrates, and a high reliance on toxic organic solvents [11,12,13,14]. Moreover, maintaining the long-term stability and functional robustness of these coatings within the dynamic, aqueous environment of the human body remains a formidable challenge.

To overcome these structural and processing bottlenecks, researcher attention has increasingly shifted toward biomimetic approaches, drawing inspiration from natural systems that effortlessly manage interfacial challenges [15,16,17,18,19,20,21,22,23,24]. In marine ecosystems, sessile organisms such as mussels and sandcastle worms (*Phragmatopoma californica*) demonstrate an extraordinary ability to achieve rapid, robust, and reversible adhesion to various wet, fouled, and chemically diverse substrates under turbulent and high-salinity underwater conditions [25,26,27]. Biochemical analyses of their secreted adhesive proteins have revealed that this superior underwater adhesion is mediated by a sophisticated two-step mechanism, which involves an initial fluid–fluid phase separation known as coacervation that allows the adhesive to displace boundary water layers and wet the substrate, followed by subsequent oxidative crosslinking typically driven by catechol chemistry [28,29,30]. By mimicking the initial fluid-like adaptation of these natural underwater adhesives, biomaterial scientists have begun to develop innovative coating platforms that can seamlessly spread uniformly across target surfaces without the need for harsh external stimuli [31,32,33].

From a biomaterial’s perspective, coacervate-based coatings offer profound advantages and unparalleled versatility for advanced medical applications [34,35,36]. Because the coacervation process occurs spontaneously in purely aqueous environments under physiological pH and temperature, it eliminates the risk of organic solvent toxicity, making it intrinsically biocompatible [36,37]. Furthermore, the dense, mesh-like network of the coacervate phase serves as an exceptionally hospitable matrix for the high-efficiency encapsulation of fragile therapeutics, including growth factors, therapeutic proteins, nucleic acids, and small-molecule drugs, shielding them from enzymatic degradation without causing denaturation [38,39,40,41]. This dual capability of simultaneously modifying the physical surface properties while acting as a localized reservoir for bioactive molecules opens up new frontiers in designing smart, stimuli-responsive drug delivery systems, anti-biofouling surfaces, and tissue engineering scaffolds [34,37,40]. However, since liquid coacervates are inherently metastable and highly sensitive to environmental fluctuations, such as shifts in pH or ionic strength, transforming these fluid-like macro-phases into long-term stable, solid-state coatings through post-coacervation curing or chemical fixation remains a critical prerequisite for their practical implementation [40,42].

Despite the growing interest in biomimetic coacervation, existing reviews predominantly focus on isolated material formulations or specific application niches, leaving a critical gap in systematically connecting fundamental processing behavior with multi-scenario clinical efficacy. This review addresses this unresolved gap by providing a targeted and comprehensive evaluation that explicitly bridges coating fabrication mechanics including thermodynamic or kinetic principles, coacervate rheology, and crosslinking strategies with full-spectrum biomedical translational scenarios. Specifically, we outline how liquid–liquid phase separation dynamics dictate interfacial adhesion and stability, and sequentially highlight recent breakthroughs across advanced biomedical fields, such as targeted drug or nucleic acid delivery, tissue engineering scaffolds, biofilm infiltration, and regenerative biointerface design. Through this integrative lens, this review establishes a clear roadmap differentiating our framework from the prior literature and rationalizing coacervate coating design for translational clinical applications.

## 2. Literature Review

Given the extensive scope and multidisciplinary nature of coacervate coating research spanning biomaterials, tissue engineering, regenerative medicine, and pharmaceutical applications, a comprehensive review of the entire body of literature is neither feasible nor practical within a single manuscript. To ensure focus, high relevance, and currency, this review systematically analyzes literature published between 2020 and 2025. To establish a rigorous and reproducible search process, comprehensive literature querying was conducted across major academic databases, including Web of Science, PubMed, and Scopus. Search queries were constructed using Boolean operators (AND, OR, NOT) combining core topic keywords.

Specifically, the exact search string applied across the database title, abstract, and keyword fields was as follows: (“coacervate*” OR “complex coacervate*” OR “coacervation”) AND (“coating*” OR “surface modification” OR “bio-interface” OR “thin film”) AND (“biomaterial*” OR “tissue engineering” OR “regenerative medicine” OR “pharmaceutical*” OR “drug delivery”) NOT (“food” OR “environmental remediation”).

Articles retrieved from the initial search were evaluated against predefined eligibility criteria.

Inclusion Criteria: (i) Peer-reviewed original research and review articles published in English between 2020 and 2025; (ii) studies explicitly focusing on bioinspired coacervate-based surface coatings, bio-interfaces, or thin-film modifications; and (iii) papers presenting clear mechanistic insights, novel formulations, or practical biomedical/pharmaceutical applications.

Exclusion Criteria: (i) Conference abstracts, patents, editorials, and non-peer-reviewed preprints; (ii) studies where coacervates were utilized solely for bulk phase separation, microencapsulation in food science, or environmental remediation without a direct focus on surface coating/interface engineering; and (iii) articles with non-accessible full texts despite institutional access efforts.

Following initial automated deduplication, titles and abstracts were systematically screened, followed by full-text evaluations aligned with our predefined thematic sections.

## 3. Fundamentals of Coacervation for Surface Coating

### 3.1. Definition and Relevance of Coacervation

Coacervation is a liquid–liquid phase separation (LLPS) process in which macromolecules partition into a dense, polymer-rich phase and a surrounding dilute phase [37,43,44,45]. In biomaterial systems, coacervates are generally classified into simple coacervates, which are formed by desolvation of a single macromolecular component, and complex coacervates, which are formed through associative interactions between oppositely charged polymers, peptides, or proteins [46,47]. Unlike precipitated complexes, coacervates retain a hydrated and fluidic character. This allows them to deform, spread, and conform to rough, wet, and chemically heterogeneous surfaces [48,49,50].

From a coating perspective, coacervation is valuable because it produces a condensed phase that remains processable after formation [48,49]. The dense phase can enrich polymers, peptides, proteins, adhesive motifs, or bioactive cargo within a confined volume while still preserving deformability and interfacial mobility [50,51]. This combination distinguishes coacervate-based coatings from conventional solvent-cast or particle-based coatings, in which surface adaptation often depends on evaporation, permanent curing, or external pressure. In coacervate-based systems, coating formation can occur under mild aqueous conditions, which is advantageous for biological applications involving proteins, cells, soft tissues, or bioactive surfaces [52,53].

A notable advantage of coacervation is the spatial and temporal separability of material deposition and subsequent stabilization [54,55]. A coacervate may first form as a mobile, interracially adaptive liquid-like phase and later be stabilized through crosslinking, ionic reorganization, oxidation, dehydration, or membrane formation [56,57]. This two-step process resembles biological adhesive systems, in which the initially secreted material is not yet in its fully hardened state [40,55,58]. For biomedical coating design, this decoupling allows surface delivery and final performance to be tailored independently at distinct stages. The material can be optimized initially for spreading and interfacial contact, followed by subsequent modifications to enhance mechanical durability, barrier functions, or biological activity [49,51,53].

Coacervation also provides a broad material platform that includes synthetic polymers, natural polysaccharides, peptide assemblies, intrinsically disordered protein systems, and hybrid organic–inorganic condensates [40,54,55,58,59,60,61]. This breadth is important because coacervation is not limited to a single chemistry or application field. Instead, it can be adapted to implant coatings, wound-contacting materials, mucosal delivery interfaces, bioadhesives, and regenerative coatings [48,49,50]. For this reason, coacervation is increasingly considered not only as a physicochemical phenomenon but also as a useful design principle for biomedical surface engineering.

### 3.2. Physicochemical Driving Forces of Coacervation

**Figure 1:**
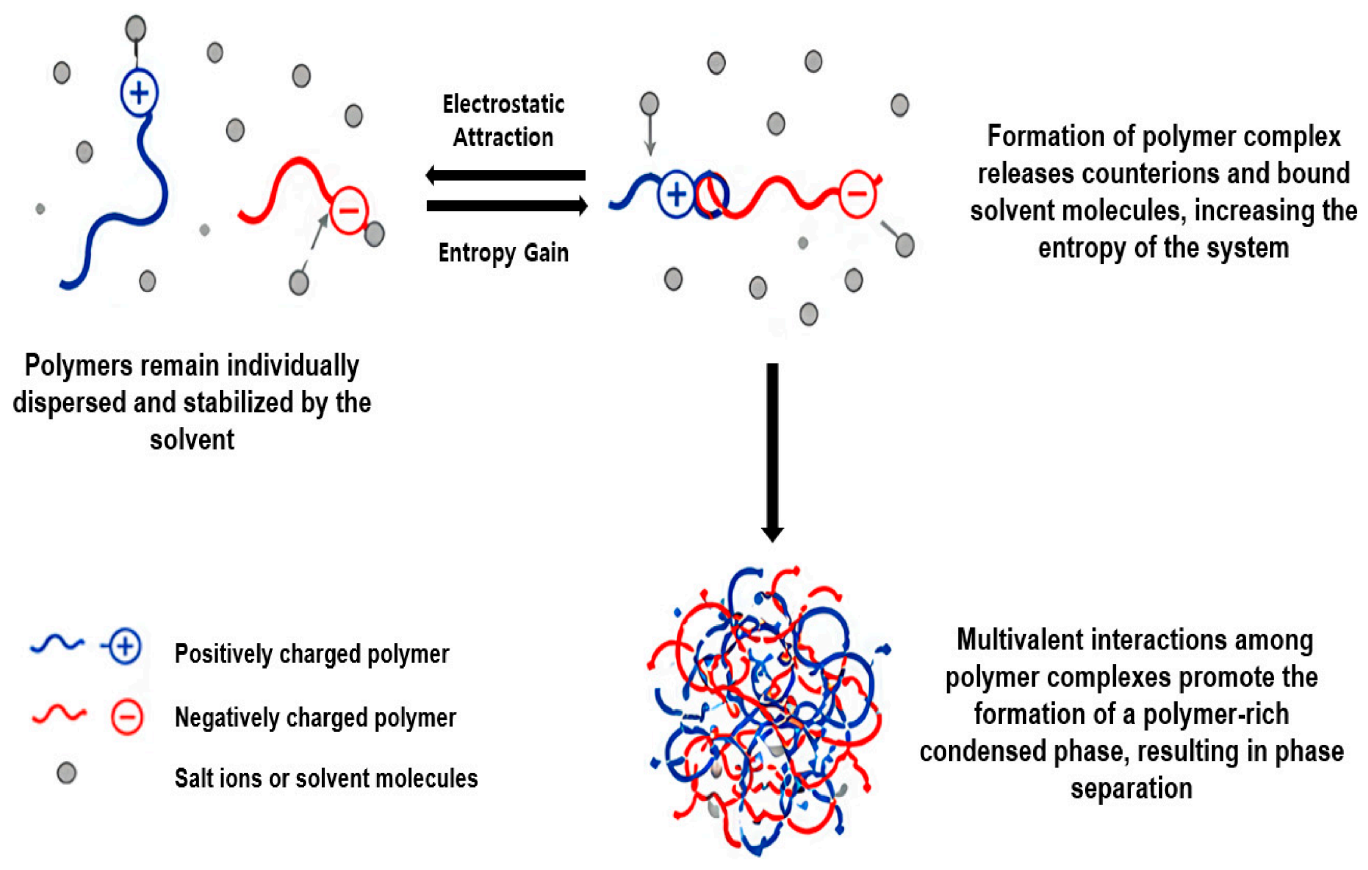
Schematic illustration of the fundamental process and mechanism of complex coacervation.

Coacervate formation is mainly governed by non-covalent interactions. In classical complex coacervate systems, electrostatic attraction between oppositely charged macromolecules is the primary driving force, while counterion release provides an important entropic contribution to dense-phase formation [54,59,60] (Figure 1). The balance among attractive interactions, translational entropy, and solvent-mediated effects is central to associative phase separation, especially in polyelectrolyte and polyampholyte systems [62,63,64]. In practical terms, coacervation occurs when the formation of a dense phase lowers the overall free energy of the system by allowing favorable macromolecular interactions to outweigh the entropic and mixing penalties of condensation.

However, electrostatic interactions alone do not fully explain coacervate behavior or its resulting surface coverage. Secondary short-range interactions including hydrogen bonding, hydrophobic association, cation–π, and π–π interactions directly dictate phase boundaries, dense-phase liquidity, and interfacial energy [37,65,66]. In peptide- and protein-based systems, the aromatic amino acid content and sequence arrangement modulate molecular cohesion and phase behavior [67,68]. For instance, incorporating tyrosine-rich motifs or polyphenol crosslinking networks significantly increases non-covalent aromatic interactions, lowering the ultra-low interfacial tension to the order of $10^{-2}\;\mathrm{to}\;10^{-1}\;\mathrm{mN}/m$ and enhancing long-term material stability against environmental degradation [37,65,66]. This reduced interfacial tension promotes spontaneous underwater wetting, decreasing contact angles (θ) on diverse substrates to below $20^{\circ }$. Conversely, an overconcentration or imbalance of these short-range interactions can trigger liquid-to-solid transitions, shifting the material from a flowable condensate to an arrested viscoelastic gel or solid aggregate [67,68]. This transition restricts interfacial mobility, ultimately yielding non-uniform, island-like coatings rather than smooth, continuous films.

Furthermore, the dense coacervate phase is not compositionally identical to the starting solution. Water, salt, counterions, and macromolecules partition unequally between the condensed and dilute phases [34,36,69,70,71]. Consequently, practical coating parameters such as film thickness, mass deposition, and topological defect density correlate directly with the local microenvironment of the dense phase rather than nominal bulk stoichiometries. Salt partitioning and excipient doping alter internal water content and macromolecular packing density, directly modulating dense-phase viscosity and volume fraction [34,36,69,70,71]. When operating under stoichiometric charge equivalence or optimized excipient ratios, dense-phase compaction is maximized, allowing the formation of uniform, pinhole-free thin coatings (20~100 nm) with low surface roughness ($R_{a}<2\;\mathrm{nm}$) [34,36,69,70,71]. Deviations in salt partitioning or ionic strength alter dense-phase hydration state and internal osmotic pressure, leading to swollen, mechanically weak films susceptible to fluid-shear delamination [34,36,69,70,71].

### 3.3. Key Parameters Governing Coating-Oriented Coacervation

The coating performance of coacervates depends strongly on formulation variables. Among these, pH is one of the most influential because it controls the ionization state of functional groups and therefore modulates electrostatic attraction [72]. Small pH changes can alter charge asymmetry, coacervate yield, droplet formation, and the transition between soluble complexes, liquid-like condensates, and insoluble aggregates. This is especially important in biomacromolecular systems, where pH can also affect protein conformation, peptide association, and cargo stability [37].

Ionic strength is another decisive factor because it screens electrostatic interactions, alters salt partitioning, and shifts the phase boundary [73,74]. In low-salt environments, electrostatic attraction is generally stronger, whereas elevated ionic strength may reduce condensation or change dense-phase viscoelasticity. This has direct relevance to biomedical surfaces, which often encounter saline conditions, serum proteins, wound exudate, or mucus. A formulation that behaves ideally in deionized water or low-ionic-strength buffer may soften, partially dissolve, or lose retention under physiologically relevant conditions.

Charge density and charge patterning also have strong effects on coacervate behavior [66,75,76]. Higher effective charge density often promotes phase separation and increases dense-phase cohesion, but it may also narrow the processing window and increase sensitivity to pH and salt. Meanwhile, charge patterning influences local association strength, chain mobility, and internal structuring. From a coating perspective, these parameters determine whether the coacervate forms a uniform interfacial layer or remains too weakly associated, too locally clustered, or too rigid for homogeneous coverage.

Molecular weight and molecular architecture further shape the behavior of coacervates during coating formation [77]. Longer chains may broaden the coacervation window, increase viscosity, and enhance interfacial persistence, but they may also reduce processability and limit spreading. Branched, comb-like, or otherwise architecturally modified polymers may alter the accessibility of charged groups and thereby influence both phase behavior and film formation. This is particularly relevant in biomedical coatings because injectability, spreadability, and post-deposition retention often need to be balanced within a single formulation.

Total concentration and mixing ratio exert complementary influences on the phase behavior. Specifically, the total concentration dictates whether the system transcends the phase boundary, whereas the mixing ratio between oppositely charged components modulates stoichiometry, dense-phase composition, and residual functional groups destined for secondary interactions [78,79]. Near charge balance, coacervation is often favored. However, deviations from stoichiometry may be useful when interfacial binding, cargo interaction, or post-coating stabilization requires excess functional groups. Ultimately, effective coating design relies not on maximizing dense-phase condensation alone, but on navigating the trade-offs between phase separation, interfacial adaptation, retention, and biological compatibility.

**Figure 2:**
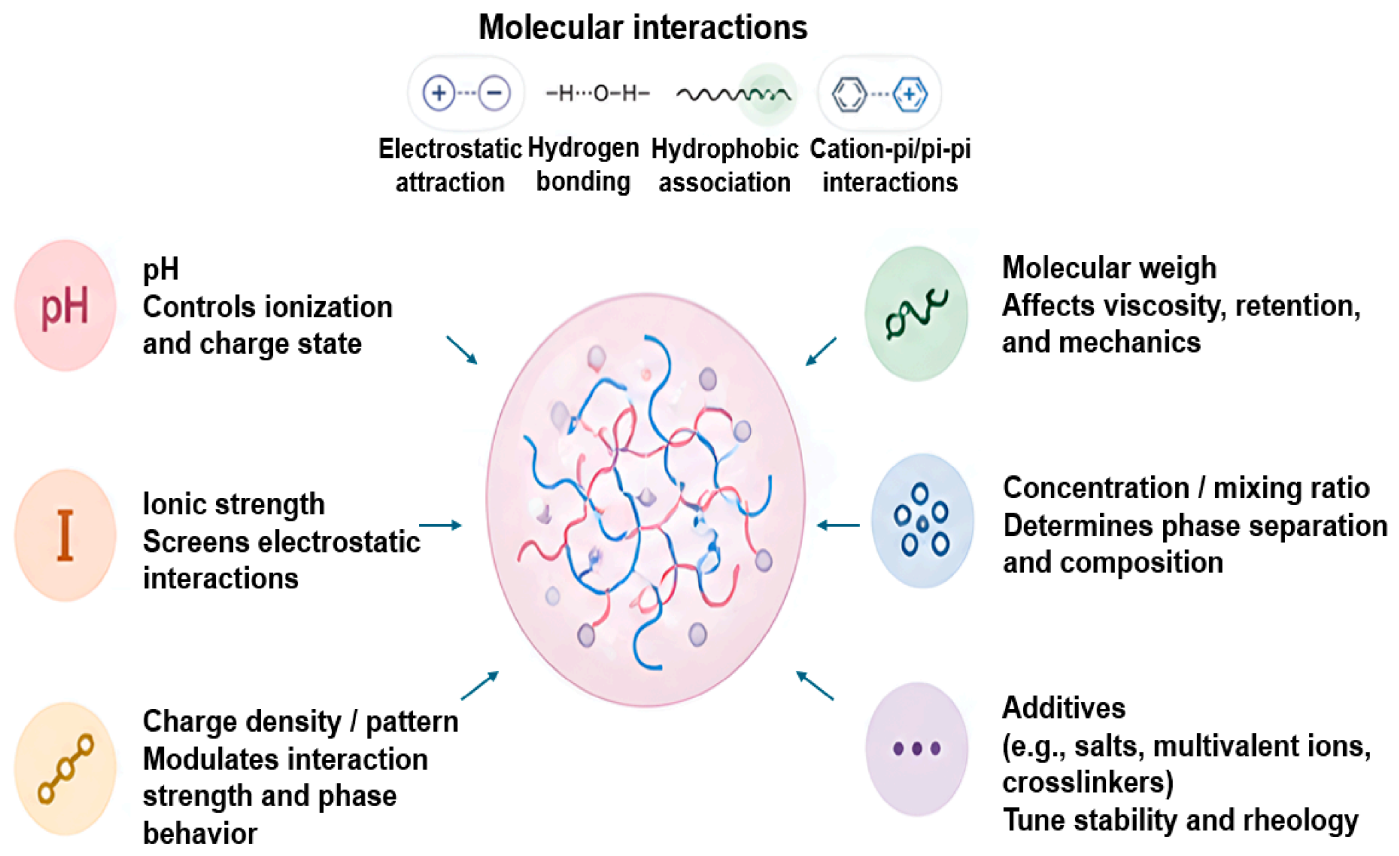
Mechanism and governing factors of coacervation-enabled surface coating. Governing Factors and Performance: Coacervate behavior is regulated by solution conditions, molecular interactions, and polymer properties.

As illustrated in Figure 2, mastering this multidimensional parameter space requires a holistic approach rather than optimizing individual variables in isolation. Because these physicochemical factors, which range from molecular architecture to environmental stimuli, are deeply interconnected, establishing a predictive framework for their synergistic effects is crucial. Balancing these trade-offs effectively transforms coacervation from a simple phase-separation phenomenon into a highly tunable, programmable platform for advanced functional coatings.

### 3.4. Interfacial Features and Bioinspired Significance

A primary merit of coacervate systems lies in their exceptional interfacial behavior, characterized by ultralow interfacial tension, high deformability, and viscoelastic flow. Specifically, polyelectrolyte complex coacervates exhibit ultralow interfacial tensions (γ) on the order of 10^−4^ to 10^−1^ mN/m, as measured by deformed drop retraction techniques [80,81]. This remarkably low interfacial energy arising from a bicontinuous fluid structure with low cohesive energy density drives rapid spontaneous wetting kinetics, allowing coacervate microdroplets to achieve complete surface coverage (>98%) within minutes across highly hydrated and irregular micro-topographies [40,66,80]. Diverging fundamentally from rigid colloids or dry coatings, liquid coacervates actively flow into surface micro-cavities without generating pinholes or stress-concentrating defects. Consequently, this dynamic adaptation provides high work of adhesion ($W_{a}$~10–50 J/m^2^) against wet, chemically heterogeneous biological interfaces, which is essential for stable coating retention under physiological flow conditions.

The rheological profile of coacervates is equally pivotal, typically manifesting as a flowable yet highly cohesive condensed phase that undergoes rapid stress relaxation under shear while retaining local structural integrity post-deposition [81,82]. Linear viscoelastic characterization reveals that the zero-shear viscosity ($\eta_{0}$) and relaxation times of coacervates can be systematically tuned across several orders of magnitude (e.g., viscosity ranging from 0.1 to 100 Pa⋅s) by varying polymer chain length, salt concentration, and charge stoichiometry. This tunable rheological continuum offers distinct operational advantages when applying coatings to compliant soft tissues or dynamic interfaces: under high shear rates during application (e.g., spraying or injecting), low transient viscosity facilitates rapid spreading and conformal layer formation, whereas post-deposition viscoelastic relaxation enables the network to reform internal cohesion, preventing wash-off by surrounding biofluids [81,82]. Thus, coacervates occupy a unique intermediate regime, presenting an optimized balance of liquid-like deformability and gel-like cohesion tailored for biomedical surface engineering.

Marine organisms like mussels and sandcastle worms achieve underwater adhesion through a two-step process: deploying fluidic precursors to target surfaces before curing [27]. Transcending a mere biological analogy, this coacervation process offers a universal solution for controlled macromolecular deposition on wet, ion-rich substrates [30,40]. Modern synthetic coacervate coatings replicate this evolutionary logic. Consequently, they bridge the gap between bioinspired design principles and advanced surface engineering, regardless of material choices or triggering mechanisms.

## 4. Biomaterials for Coacervate-Based Coatings

**Figure 3:**
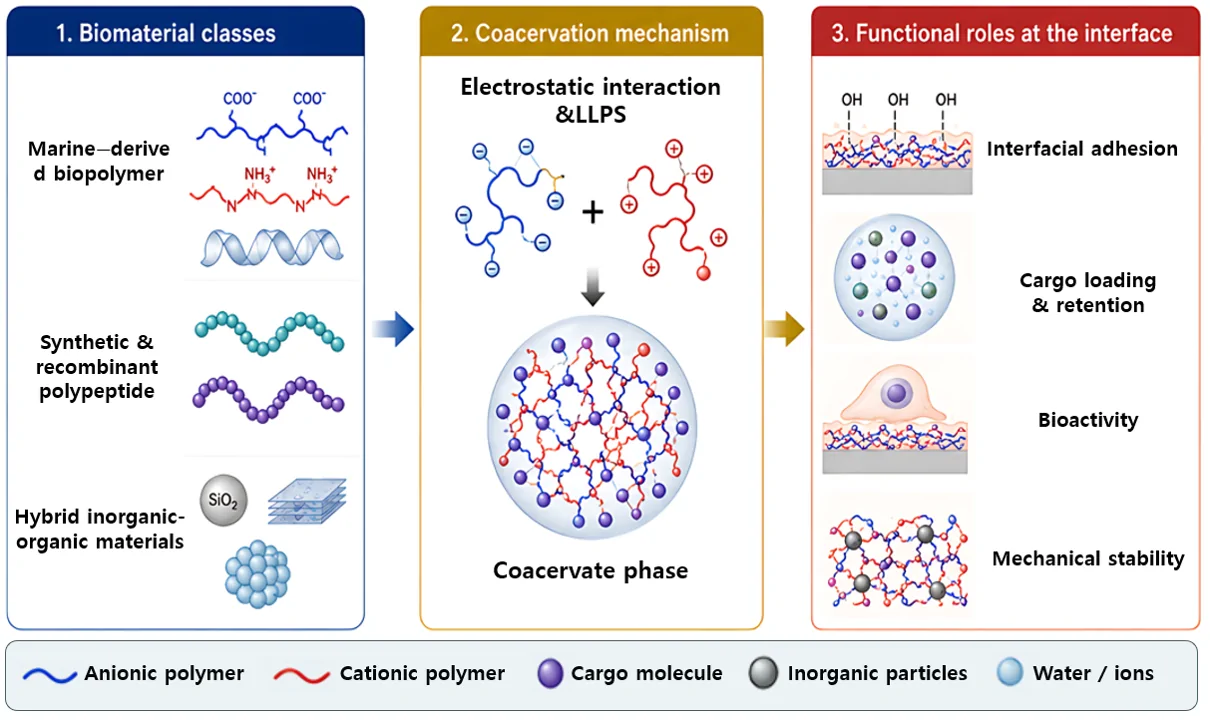
Biomaterial classes, coacervation mechanism, and interfacial functions. (1) Biomaterial classes: Marine-derived biopolymers, synthetic/recombinant polypeptides, and hybrid inorganic–organic materials used for coacervation. (2) Coacervation mechanism: Formation of a hydrated, liquid-like coacervate phase via electrostatic interactions and LLPS. (3) Functional roles: Integrated multifunctionality within a single layer, including wet adhesion, cargo encapsulation, bioactivity (cell adhesion/proliferation), and network-reinforced mechanical stability.

The translation of coacervates into functional coating platforms relies heavily on the macromolecular design and intrinsic properties of the constituent biomaterials [37] (Figure 3). The selection of these building blocks critically dictates the thermodynamic driving forces, including electrostatic interactions, hydrophobic effects, and hydrogen bonding, that govern the coacervation process [83]. Concurrently, it determines the viscoelastic, mechanical, and adhesive performance of the resulting interfacial films. In recent years, the library of biomaterials engineered for coacervate coatings has expanded significantly, evolving from conventional naturally derived polymers to sophisticated synthetic architectures capable of precise spatial and temporal control at the substrate interface [36,83].

A central consideration in biomaterial selection is that each component can perform multiple, interdependent functions at the interface rather than serving a single isolated role [41,84]. Polycaccharide may provide electrostatic functionality for coacervate formation while simultaneously regulating hydration, modulating interfacial viscosity, and influencing cargo partitioning within the condensed phase [85,86]. Recombinants or sequence-defined proteins can similarly contribute to the thermodynamics and kinetics of phase separation while presenting adhesive domains or bioactive motifs that regulate cell–material interactions [87,88]. Inorganic or mineral phases may reinforce the coating while providing biological cues such as mineralization, UV shielding, antibacterial effects, or ion-mediated signaling [89,90,91,92]. Thus, coacervate-based coatings can integrate structural, adhesive, and biological functions within a single interfacial layer.

### 4.1. Marine-Derived Biopolymers

Marine-derived biopolymers are attractive for coacervate-based coatings because they combine biological compatibility, abundant ionizable groups, and mild aqueous processability [41,93]. Their importance lies not only in their natural origin but also in their structural and chemical suitability for hydrated interfacial applications [36,84]. Many marine polysaccharides and proteins can participate in electrostatic association, hydrogen-bonding networks, or ionic crosslinking, all of which support the formation, deposition, and stabilization of coating-relevant condensed phases [84,93].

Alginate is a representative anionic marine-derived polysaccharide that is widely used because of its carboxyl-rich structure and its ability to interact with cationic species and multivalent ions [85,94]. In coacervate-related coating systems, alginate can function both as a phase-forming component and as a post-deposition stabilizing component. It can participate in associative interactions with cationic species and can subsequently be fixed, at least in part, through ionic gelation or secondary stabilization [85,95]. This provides a convenient route from interfacial condensation to coating persistence, particularly when bioactive cargo protection is also required [94,95]. Such dual functionality is particularly valuable in biomedical coating systems, where mild processing is often required but long-term interfacial retention remains desirable [85,94].

Chitosan is another highly relevant material because of its cationic nature, biodegradability, antimicrobial potential, and capacity for pH-dependent complexation with anionic polymers or proteins [45,86,96]. In coacervate systems, chitosan is far more than a simple positively charged partner for polyanions, as it actively dictates phase behavior, rheology, and post-deposition cohesion [45,96]. This multifunctional role renders it exceptionally well-suited for wound and mucosal applications, where coatings must adhere to wet biological surfaces while maintaining local retention and bioactivity [86,96]. In wound-contacting coatings, for example, the ability of chitosan-containing coacervates to combine condensation behavior with antimicrobial and wet-adhesive functions can be particularly advantageous [45,86].

Marine collagen and gelatin provide ECM-like bioactivity and regenerative relevance, especially for tissue-contacting applications [86,94]. These materials are attractive when the coating is expected to do more than simply remain on the substrate, because proteinaceous coacervate components can also support hydration, biomolecule partitioning, and cell-relevant interactions [85,86]. Marine collagen-derived gelatin is of particular interest because fish gelatin can act as a charged protein partner in complex coacervation while retaining collagen-derived biological relevance [86,96]. Gelatin generally provides greater formulation flexibility than native collagen in aqueous coacervate systems, especially when it is paired with oppositely charged polysaccharides such as alginate or carboxylated chitosan [85,86]. Their main limitations include modest mechanical robustness and sensitivity to hydration and temperature, which often require blending, condensation with oppositely charged partners, or secondary stabilization after deposition [85,95].

Marine-origin and marine-inspired polyanions or polyelectrolytes further expand the design space of coacervate coatings by providing tunable charge density, cargo affinity, and tissue-specific interactions [97]. These materials can be especially useful when the coating must function simultaneously as a barrier, a bioadhesive layer, and a cargo reservoir. Therefore, marine-derived biopolymers should be recognized as active functional materials rather than passive matrices. Their distinct chemistry dictates the entire coating lifecycle, governing key processes from coacervate formation and interfacial retention to ultimate biological performance.

### 4.2. Synthetic and Recombinant Polypeptides

Synthetic and recombinant polypeptides provide a distinct route for coacervate-based coating design because they allow for a high degree of control over charge, sequence, molecular architecture, and responsiveness [36,84]. Whereas marine-derived biopolymers are often attractive because of their biological familiarity and hydrated processability, synthetic and recombinant systems are valuable because they can be engineered more deliberately [41,84]. This distinction is especially important in coacervation research, where subtle changes in sequence patterning, aromatic content, charge spacing, or molecular flexibility can strongly influence whether a condensed phase forms and how it behaves after deposition [36,84].

Elastin-like polypeptides (ELPs) are among the most representative examples of programmable coacervate-forming materials [98,99]. Their ability to undergo environmentally responsive phase transitions makes them useful in systems where coating formation or reorganization must be triggered by pH, temperature, osmolytes, or related physicochemical cues. For coacervate-based coatings, ELP-derived systems are not simply passive structural materials because they can serve as programmable components that help define when, where, and how condensation occurs [99,100]. This makes them especially attractive for applications requiring reversible assembly, triggerable deposition, or adaptive interfacial behavior. In addition, ELPs can be engineered to alter charge, hydrophobicity, and responsiveness, allowing them to function as tunable condensate-building blocks rather than fixed biomaterials.

Recombinant mussel-inspired proteins are particularly significant in this regard because they bridge marine adhesive inspiration and sequence programmability [87,101,102]. They are well suited for biomimetic coatings designed for wet biological surfaces because they can combine coacervate formation with wet adhesion and bioactive interfacial interactions [87,102]. Hyaluronic acid/recombinant mussel adhesive protein coatings on titanium promote osteoblast proliferation as an example of coacervation-based coatings [102]. This study is notable because it demonstrates how condensed interfacial coating can combine wet adhesion, soft deposition, and biological performance. More broadly, recombinant mussel-inspired proteins allow adhesive motifs, catechol-related functionality, and tailored charge distribution to be incorporated into a single material-design framework.

### 4.3. Hybrid and Inorganic-Reinforced Coacervate Materials

Hybrid coacervate materials are increasingly important because many biomedical coatings must do more than wet and remain at the interface [41,93]. They may also need to resist deformation, reinforce a substrate, provide mineral-related bioactivity, regulate transport, or preserve structural integrity under prolonged exposure to aqueous environments [60]. Purely soft coacervates are often excellent for interfacial adaptation, but this softness can become a limitation when coatings are used on implants, mechanically challenged tissues, or mineralized interfaces.

Silica is one of the most direct examples of an inorganic component incorporated into a hybrid complex coacervate [60]. Silica-containing hybrid coacervates can alter rheology, cohesion, and condensed-phase organization, thereby influencing not only the mechanical stability of the final coating but also its processability during deposition. This is an important distinction because the inorganic component does not simply strengthen the coating after deposition. Instead, it may influence the coacervate itself, including how densely it forms, how it flows, how it wets the substrate, and how it reorganizes after deposition.

Integrating inorganic nanoclusters into complex coacervate matrices formed via LLPS has emerged as a powerful strategy to overcome structural limitations and impart novel functionalities to hybrid materials. In particular, polyoxometalates (POMs), which are polyatomic anionic metal oxide clusters, have attracted significant attention as inorganic reinforcing agents and functional building blocks [103]. Organic–inorganic POM hybrid networks possess exceptional structural stability. This stability is driven not merely by basic electrostatic attractions but by a sophisticated network of non-covalent interactions, including coordination bonding, hydrogen bonding, and π–π stacking. This structural tunability and physicochemical versatility effectively overcome the intrinsic kinetic instability and poor mechanical strength of conventional polymer-based coacervates, laying a solid groundwork for advanced stimuli-responsive nanomaterials.

Translating these inorganic reinforcement principles to biomimetic systems, the co-assembly of short peptides with polyanionic POM nanoclusters (e.g., Keggin-type structures) spontaneously triggers LLPS in aqueous media [104]. This hybrid system exhibits remarkable multi-responsiveness driven by distinct chemical triggers. Alterations in external pH modulate the ionization states of peptide residues to reversibly drive phase transitions, whereas transition metal ions induce localized coordination with chelating residues (such as imidazole rings) to dynamically enhance mechanical strength and alter condensation behavior. Consequently, short peptide/POM hybrid coacervates hold profound potential for applications demanding precise dynamic control in aquatic or biological environments, including stimulus-responsive underwater adhesives, biomimetic protocell models, and localized micro-reactors.

The integration of inorganic precursors into coacervate frameworks has opened new avenues for advanced biomedical applications, particularly in developing self-mineralizing bone tissue scaffolds and hard-tissue adhesives [89]. Cationic coacervates can serve as highly efficient phosphate ionic reservoirs that fundamentally modulate the nucleation and growth kinetics of calcium phosphates. By localizing dense concentrations of phosphate ions within the phase-separated liquid matrix, these macromolecular assemblies effectively regulate the precipitation pathway of mineral phases from amorphous precursors to crystalline structures. This controlled mineralization capability transforms the traditionally weak organic coacervate into a mechanically robust composite, mimicking the natural process of biomineralization and offering a highly tunable platform for hard tissue engineering.

Building upon these mineralizing reservoirs, recent breakthroughs have successfully translated coacervate systems into multifunctional injectable underwater adhesives tailored for critical bone defect restoration and cranial flap fixation. For instance, visible light-induced simultaneous bioactive amorphous calcium phosphate mineralization combined with in situ crosslinking strategies yields highly cohesive, injectable hydrogels that exhibit exceptional underwater adhesion strength and significantly accelerated bone regeneration [91]. Similarly, advanced wet adhesive and antibacterial poly(allylamine hydrochloride) and tripolyphosphate coacervates with intrinsic self-mineralizing capabilities have been engineered to address the stringent requirements of cranial bone stabilization [92]. These hybrid systems not only maintain strong mechanical fixation in wet physiological environments but also actively release bone forming ions and suppress bacterial colonization. Consequently, these self-mineralizing and crosslinkable coacervate adhesives represent a major milestone in translational biomaterials, offering powerful synergistic solutions that combine structural tissue sealing with active osteogenesis.

Each biomaterial class presents distinct trade-offs: marine biopolymers offer cost-effective biocompatibility but weak mechanical strength, recombinant polypeptides provide precise sequence-defined coating uniformity at higher costs, and inorganic hybrids deliver superior mechanical reinforcement with increased processing complexity (Table 1). Consequently, balancing these material-specific advantages is essential for designing target-specific coacervate coating platforms.

## 5. Coating Strategies and Methodologies

**Figure 4:**
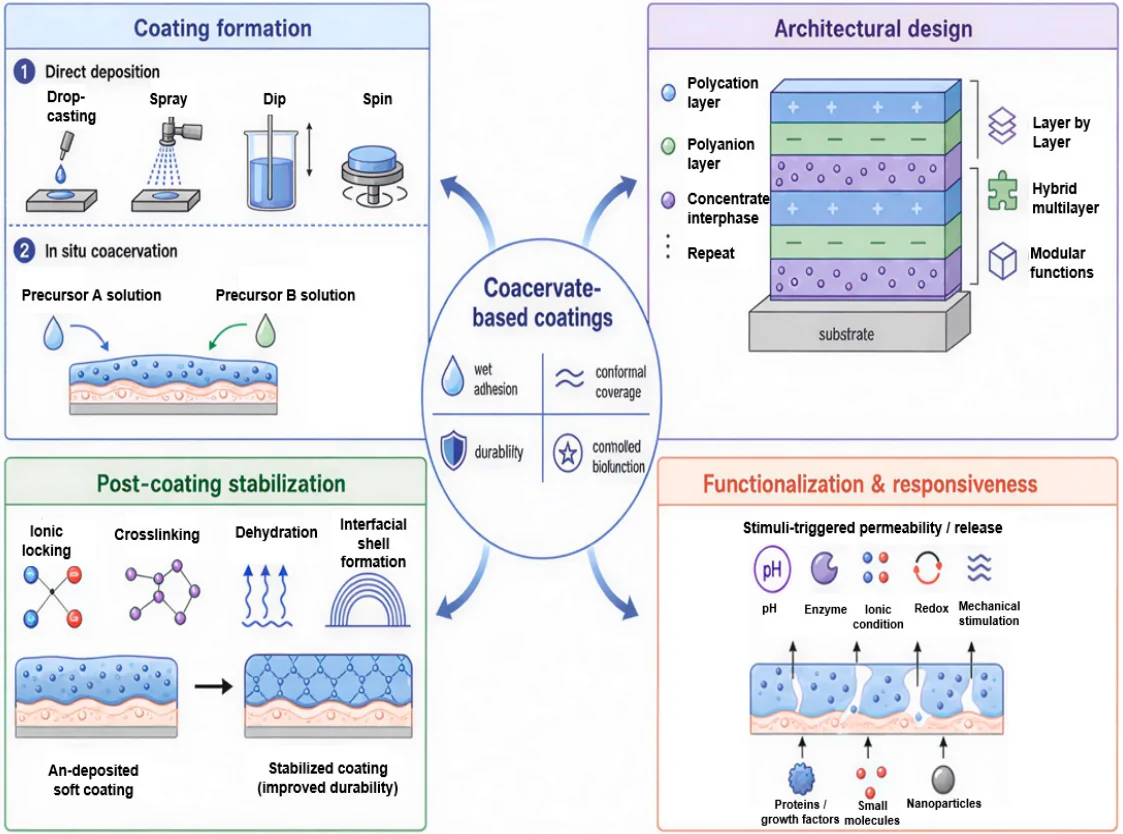
Overview of the fabrication, structural orchestration, and functionalization paradigms of coacervate-based interfacial coatings. Centered around their inherent traits (wet adhesion, conformal coverage, durability, and bioactivity), the developmental workflow is divided into four main axes: (i) Coating formation: Direct deposition of preformed coacervates using methods such as drop-casting, spraying, dip-coating, and spin-coating, as well as in situ coacervation driven by mixing precursor solutions (precursors A and B) directly on the target substrate. (ii) Architectural design: Layer-by-layer (LbL) assembly and hybrid multi-stratified systems composed of alternating polycation (blue spheres) and polyanion (green spheres) layers, separated by concentrated coacervate interphases. (iii) Post-coating stabilization: Transition from soft, as-deposited coacervate coatings to highly durable stabilized coatings via secondary interactions, including ionic locking, chemical crosslinking, dehydration, and interfacial shell formation. (iv) Functionalization & responsiveness: Stimuli-responsive gating and cargo release behavior (e.g., proteins/growth factors, small molecules, and nanoparticles) triggered by environmental cues such as pH changes, enzymatic activity, ionic conditions, redox potential, and mechanical stimulation.

Surface coating technologies utilizing complex coacervation provide a powerful platform for selectively modifying the physicochemical properties of surfaces by precisely controlling electrostatic interactions and phase separation phenomena between polymers [40,54,55,58,60]. To stably form a coacervate-derived coating layer on a target substrate and maximize its functionality, it is essential to establish an optimal processing strategy tailored to the surface characteristics, the required film thickness, structural uniformity, and the ultimate application objectives [48,49,50,51,52,53]. This chapter provides various established methodologies, ranging from the direct deposition of preformed coacervates onto substrates to in situ coacervation processes that induce real-time phase separation at the target interface (Figure 4).

### 5.1. Direct Deposition of Preformed Coacervates

A highly intuitive and widely adopted approach involves the direct deposition of a preformed dense phase onto the target substrate. Depending on viscosity and wetting behavior, this may be achieved by dip-coating, drop-casting, painting, spray-assisted deposition, or spin-coating [105,106]. Among these, spin-coating is useful for relatively thin and uniform films, whereas painting or casting is more suitable for localized coverage of irregular wet surfaces. Direct deposition is attractive because it reduces processing steps and allows faster build-up of coatings when the condensed phase retains sufficient flowability and cohesion [107,108].

A major practical advantage of this method is that the dense phase can be characterized before it ever reaches the substrate. Its viscosity, spreading behavior, and cargo content may be optimized in advance, which can be useful for reproducibility and formulation screening. This is especially relevant in early-stage biomaterial development, where the ability to decouple formulation optimization from application variability can save considerable effort [106,107,109,110]. Spin-coating of polyelectrolyte complex coacervates produces smooth, uniformly thin coatings whose thickness can be tuned through salt concentration and processing conditions [105]. Expanding into biomedical applications, Miller et al. utilized mussel-derived complex coacervates to form lubricating interfacial layers, thereby mitigating frictional surface damage. This highlights the distinct advantage of this strategy, where a preformed dense phase can be fully characterized and optimized prior to surface application [107].

However, preformed dense phases also present practical limitations. Because condensation already occurs before contact with the surface, the system may be sensitive to handling history, dilution, air exposure, or shear [109]. A coacervate that appears ideal in bulk may spread poorly or unevenly when confronted with a specific hydrated interface. In addition, certain substrates may not provide sufficient affinity to retain the condensed phase unless secondary fixation is applied rapidly. Thus, direct deposition is most effective when dense-phase rheology, substrate chemistry, and post-deposition stabilization are optimized together rather than sequentially [106,108,110]. From a practical processing perspective, the scalability and geometric compatibility of each method emerge as critical limiting factors [105,106]. Spin-coating is highly useful for flat substrates and relatively uniform thin films, but it is less suitable for highly irregular or spatially restricted biological surfaces. In contrast, drop-casting or painting may be better for localized interfacial coverage but may produce greater thickness heterogeneity. Therefore, direct deposition is not a single method but a family of methods whose usefulness depends strongly on the target interface and performance goal.

From a quantitative processability standpoint, the choice among direct deposition techniques dictates film thickness and throughput. Spin-coating offers precise nanoscale-to-microscale thickness control (~10 nm to 5 µm), governed primarily by angular velocity (rpm) and solution viscosity; however, its batch throughput remains limited to single-substrate processing (<60 units/h). Conversely, dip-coating accommodates broader thickness profiles (~50 nm to 50 µm), where thickness scales with withdrawal speed following the Landau–Levich regime ($h\propto \eta^{2/3}v^{2/3}$), enabling high-throughput, roll-to-roll continuous manufacturing at lower relative equipment costs. Spray-assisted deposition further expands throughput across large-area substrates (100 nm to 100 µm), offering an economical route for industrial-scale encapsulation despite higher thickness variability [105,106].

### 5.2. In Situ Coacervation at the Target Interface

Coacervation may also be induced directly at the substrate or tissue interface rather than being formed beforehand. In such systems, precursor solutions are transformed into a condensed coating layer by local changes in pH, ionic strength, redox state, or interfacial mixing. This approach is particularly useful for wet, curved, porous, or bleeding surfaces, because it improves conformal contact and is compatible with minimally invasive delivery [61,111,112,113,114,115].

Initially, the fundamental mechanism of salt-induced phase separation at the interface was demonstrated through the interplay of electrostatic and non-covalent forces. Kim et al. [114] revealed that simple coacervation of underwater adhesives can be triggered by the high ionic strength of seawater. Specifically, when the adhesive sequences are exposed to saline conditions, the shielding of electrostatic repulsions allows cations to interact dynamically with aromatic π electrons (“cation-” π interactions). This salt-triggered in situ coacervation enables the adhesive to displace hydration layers on the target surface, allowing the coacervate to wet the substrate effectively and initiate strong interfacial adhesion.

The conceptual strength of in situ coacervation lies in the fact that the material is assembled in the same environment in which it is expected to function. This reduces the need to transfer a fragile condensed phase from one condition to another and can improve matching between phase formation and local interface properties. On tissues, this may enable better adaptation to curvature, microtopography, or fluid-covered surfaces than would be possible with a preformed coating [29,61,112,113,115,116]. Building upon these fundamental principles of interfacial coacervation, recent strategies have integrated smart stimuli to transition these liquid-like coacervates into functional, solid-state hydrogels directly at the lesion site. Yun et al. [91] expanded the utility of this phenomenon into bone tissue engineering by developing an injectable, coacervate-based underwater adhesive hydrogel. At the target interface, this system undergoes a visible light-induced dual mechanism: simultaneous bioactive amorphous calcium phosphate (ACP) mineralization and in situ covalent crosslinking. The light-mediated crosslinking rapidly solidifies the coacervate matrix at the interface, preventing dissolution and fixing the mineral phase within the hydrogel network. This coordinated in situ transition not only secures stable mechanical fixation under wet physiological conditions but also provides an osteoinductive microenvironment that significantly enhances bone regeneration.

In situ systems are especially attractive for applications such as tissue sealing, bleeding control, and injectable interfacial materials, where external coating application may be physically difficult or clinically impractical. They also allow the local environment itself to participate in coating formation, which is conceptually appealing for responsive and biomimetic materials. However, this strategy necessitates rigorous control over triggering kinetics to avoid a critical trade-off between transport and stability. Premature triggering restricts precursor delivery and target coverage, while sluggish kinetics result in the diffusion and dilution of reactive species prior to effective film consolidation [29,61,91,113,115,116].

Hence, successful in situ coacervation relies on a delicate interplay between precursor stability, trigger kinetics, and local boundary conditions. The focus must extend beyond mere thermodynamic coacervation potential to encompass the spatiotemporal synchronization of the phase transition and the optimization of the emergent dense-phase properties. This fundamental dependency makes in situ modalities extraordinarily powerful yet substantially more sensitive to precise formulation parameters than direct deposition [113,115,116].

### 5.3. Layer-by-Layer (LBL) and Hybrid Multilayer Coatings

Layer-by-layer (LBL) assembly remains a versatile route for multifunctional coatings and coacervate concepts can be incorporated into this framework through polyelectrolyte multilayers, coacervate-like interphases, or hybrid inorganic/organic layers [117]. These strategies are especially useful when a single condensed layer is not sufficient and the coating must instead provide multiple functions in a spatially organized way. The major advantage of LbL-based strategies is architectural control. Thickness, composition, sequence of assembly, and sometimes even local gradient formation can be more tightly controlled than in simple one-step deposition. This is particularly useful for biomedical coatings that must integrate several roles without compromising each one [118,119,120].

The LbL deposition technique, driven by complementary electrostatic and non-covalent interactions, offers a powerful and versatile platform for the nanoscale engineering of multifunctional hybrid multilayer coatings. Yu et al. [121] demonstrated the efficacy of this approach by developing LbL-deposited hybrid polymer coatings composed of naturally derived polysaccharides and zwitterionic silanes tailored for marine antifouling applications. By integrating the highly hydrated, fouling-resistant architecture of zwitterionic silane moieties within the structural framework of a polysaccharide matrix, their hybrid multilayer system successfully suppressed the non-specific settlement of marine organisms. In the context of advanced coatings, incorporating organic–inorganic hybrid constituents and functional polyelectrolytes into an LbL sequence reinforces the film’s mechanical durability while optimizing its physicochemical barrier properties. Consequently, this provides a robust strategy for achieving long-term protection and antifouling performance in harsh aquatic environments.

Concurrently, conventional multilayer assembly is inherently limited by substantial time and labor investment, especially during the growth of macroscopically thick films [118,119]. Coacervate-mediated hybrid approaches circumvent these drawbacks by bridging the gap between the meticulous structural fidelity of LbL assembly and the rapid, mass-transfer-efficient accumulation of dense condensed phases. The mechanical robustment and operational versatility of these multilayer films can be substantially augmented by transitioning from purely polymeric systems to inorganic–organic hybrids. Lengert et al. [118] identified nanoparticles embedded within polyelectrolyte multilayer films and capsules as key enabling components of modern hybrid coatings. The integration of zero-, one-, or two-dimensional nanostructures introduces tailored magnetic, optical, electrical, or mechanical functionalities that are unattainable with polymers alone. In a hybrid coating context, these embedded nanoparticles serve a dual purpose: they structurally reinforce the soft polyelectrolyte matrix against mechanical stress and simultaneously act as responsive centers for remote activation or signal transduction.

Quantitatively, although LbL assembly achieves unmatched architectural precision at the nanoscale (~1 nm per bilayer pair, with total thickness finely tuned up to ~1 µm), its operational throughput is intrinsically bottlenecked by repetitive dipping and rinsing cycles. This low batch capacity and high time/solvent consumption elevate relative manufacturing costs, necessitating the integration of spray-assisted or acoustofluidic LbL platforms to accelerate mass transfer and improve scalability.

Finally, advanced technological interventions have revolutionized the assembly efficiency and spatial precision of these nanoparticle-integrated LbL systems. Rowland et al. [122] detailed the mechanisms and technologies behind layer-by-layer nanoparticle assembly for biomedical applications, highlighting the transformative role of acoustofluidics in accelerating the fabrication process. Conventional LbL methods often suffer from prolonged diffusion-limited deposition times. However, the integration of acoustic fields drives rapid, uniform particle transport and alignment at the substrate interface. This synergy between advanced fluidics and nanoscale hybrid assembly establishes a highly scalable, high-throughput manufacturing paradigm for the next generation of sophisticated, multi-component functional coatings.

To provide a systematic engineering comparison, the representative coating strategies are benchmarked in Table 2 based on their quantitative parameters, including film thickness range, batch throughput, and relative manufacturing costs.

### 5.4. Post-Coating Stabilization

The long-term structural integrity and functional stability of complex coacervate-based coatings are critically challenged by their inherent thermodynamic instability and high sensitivity to environmental stimuli such as pH, ionic strength, and hydration state. To address these limitations, post-coating stabilization strategies have evolved from simple physical entrapment to sophisticated chemical and structural reinforcement networks. Zhao et al. [123] demonstrated that post-assembly covalent crosslinking of coacervate matrices significantly enhances both mechanical robustment and functional longevity. By immobilizing and stabilizing proteins within a covalently crosslinked coacervate framework, they not only prevented the leaching of active biomacromolecules but also observed enhanced enzymatic activity under stressful conditions.

To achieve superior mechanical resilience while maintaining the dense, highly hydrated nature of coacervates, the development of interpenetrating polymer networks (IPNs) has emerged as a powerful stabilization paradigm. Li et al. [124] successfully engineered polyelectrolyte complex-covalent IPN hydrogels, showcasing a dual-network approach where the reversible physical electrostatic interactions of the coacervate coexist with a permanent chemically crosslinked covalent network. When applied as a post-coating strategy, such IPN architectures provide a synergistic mechanism: the covalent mesh anchors the coating to prevent disintegration, while the dynamic polyelectrolyte complexes dissipate mechanical energy.

Beyond structural reinforcement, stabilizing coacervates in challenging dynamic environments, particularly underwater, requires specialized chemical adaptations that prevent premature dissolution and loss of adhesion. Peng et al. [125] addressed this by developing a coacervate-derived hydrogel system featuring effective water repulsion combined with robust underwater bioadhesion. In a post-coating context, inducing hydrophobic domains or secondary crosslinking within the coacervate phase after deposition can effectively expel interfacial water molecules. This water-repelling transition locks the coacervate structure firmly in place. Consequently, it transforms a highly sensitive phase into a water-resistant, adherent barrier capable of promoting tissue healing and withstanding physiological shear forces without delamination.

Furthermore, the stability of coacervate coatings must be evaluated against cyclic environmental fluctuations, such as drying and rehydration, which are common in both biomedical and environmental applications. Fares et al. [126] systematically investigated the impact of wet–dry cycling on the phase behavior and compartmentalization properties of complex coacervates. Their findings indicate that evaporation-driven concentration can trigger significant structural transitions and alter the internal partitioning of encapsulated species.

Finally, precise control over the external boundary of the coacervate coating is essential to prevent unintended coalescence or degradation triggered by surrounding bulk fluids. Yin et al. [127] demonstrated that engineering the coacervate microdroplet interface via polyelectrolyte and surfactant complexation provides an elegant pathway for interfacial stabilization. By forming a structured molecular shield at the interface, this approach effectively suppresses macroscopic phase separation and stabilizes individual coacervate domains against environmental fluctuations. Integrating interfacial engineering with internal chemical crosslinking provides a comprehensive, multi-scale stabilization strategy. This dual approach ensures that coacervate coatings maintain both their surface-level functionality and overall structural fidelity over extended operational lifetimes.

### 5.5. Stimuli-Responsive Coatings

A defining technological merit of coacervate-based coatings lies in their intrinsic adaptability to environmentally responsive architectures. In response to local biological conditions, the dense phase can respond to pH, ionic conditions, enzyme activity, redox state, and mechanical stimulation to alter permeability, reorganization, or release behavior [37,128,129,130].

The integration of smart, stimuli-responsive functionalities into complex coacervate coatings represents a significant advancement in adapting these materials to dynamic environmental changes. Complex coacervates are inherently sensitive to external chemical and physical triggers due to the reversible nature of their underlying electrostatic and non-covalent interactions. Lawrence and Lapitsky et al. [128] systematically elucidated this sensitivity by examining ionically cross-linked poly(allylamine) systems, specifically demonstrating how external variations in ionic strength and pH govern phase behavior and material properties. In the context of smart coatings, leveraging these specific ion- and pH-dependent triggers allows for precise tuning of the material’s structural state. This enables a controlled transition from a cohesive, dense barrier to a responsive system that alters its physical properties on-demand within fluctuating aqueous environments.

To transition from macroscopic adhesion to targeted biochemical functionality, responsive coacervate coatings can also be engineered to respond to internal physiological triggers, such as cellular redox states. Wang et al. [129] introduced redox-responsive peptide-based complex coacervates designed as smart delivery vehicles capable of the controlled release of proteinous drugs. Incorporating such redox-active motifs into a surface coating framework introduces a valuable spatio-temporal control mechanism. Upon encountering a reduction–oxidation gradient, such as within the intracellular environment or inflamed tissue sites, the coacervate matrix undergoes controlled phase dissolution or localized mesh expansion. This structural transition triggers the precise, on-demand release of encapsulated therapeutics directly from the functionalized surface.

Ultimately, the synergy of multiple responsive behaviors within a single coating architecture enables unprecedented performance under complex, real-world operational profiles. Dompe et al. [131] demonstrated the exceptional utility of multiresponsive complex coacervates for underwater adhesion, where the coacervative phase simultaneously reacts to multiple independent triggers, such as temperature and ionic changes. For advanced coating applications, this multi-responsiveness provides a crucial buffering capacity and operational versatility. By precisely coordinating structural changes across multiple environmental coordinates, these advanced multiresponsive coacervate coatings can sustain, tune, or cleanly terminate their adhesive and protective functionalities. This dynamic adaptability establishes a highly versatile platform for soft robotics, marine coatings, and smart wound dressings [132,133,134,135,136].

To provide a structured comparison of these methodologies, the distinct technical features, strategic advantages, and inherent operational constraints of current coacervate-based coating techniques are comprehensively compiled in Table 3. As summarized, the selection of a specific coating route necessitates a careful assessment of the target interface alongside the intended performance objectives.

Overall, a comprehensive comparison of these coacervate-based coating strategies highlights distinct technical trade-offs among operational simplicity, structural durability, manufacturing scalability, and clinical translational potential (Table 3). While direct deposition and post-coating stabilization offer straightforward industrial scale-up owing to compatibility with existing batch-processing infrastructure, LbL assembly and stimuli-responsive platforms provide superior architectural control at the cost of higher manufacturing complexity. From a translational perspective, selecting the optimal fabrication route requires balancing the physical boundary conditions of the target bio-interface (e.g., static implant vs. dynamic wet tissue) with primary performance demands (e.g., rapid tissue sealing vs. sustained cargo release). Rather than seeking a single universal method, future translational design will rely on integrating these complementary approaches to tailor coacervate interfaces for specific biomedical and industrial applications.

## 6. Mechanical Characteristics of Coacervate Coatings

The mechanical characteristics of coacervate coatings play a fundamental role in determining their stability, durability, and functional performance under physiological conditions. Unlike conventional coatings, coacervate-based coatings are typically formed through reversible, non-covalent interactions, resulting in a unique viscoelastic behavior that enables strong interfacial adhesion while maintaining structural adaptability [34,40]. Consequently, understanding and precisely tailoring these mechanical profiles is of paramount importance in the biomedical realm. Complex physiological environments present demanding operational requirements for applications such as medical implants, tissue engineering scaffolds, wound healing matrices, and drug delivery systems. To function seamlessly in these settings, a synergistic integration of robust wet interfacial adhesion, mechanical strength, compliant elasticity, and long-term durability is indispensable [37].

### 6.1. Viscoelastic and Rheological Behavior

The primary mechanical hallmark of coacervate coatings lies in their transitional viscoelastic state. Coacervates exhibit distinct viscoelastic behavior, characterized by the dynamic relationship between their storage modulus (*G′*) and loss modulus (*G″*). Under high shear conditions, they display liquid-like shear-thinning properties, facilitating uniform spreading over complex, micro-structured biomaterial surfaces. Upon relaxation, they rapidly transition to a gel-like state. This liquid-to-solid transition and the accompanying self-healing capability, driven by the dynamic association and dissociation of non-covalent bonds (e.g., electrostatic and hydrogen bonding), allow the coating to spontaneously repair micro-defects or cracks caused by physical deformation in physiological environments [36].

The complex coacervate system composed of whey protein isolate (WPI), chitosan (CH), and garlic essential oil (GEO) exhibits a pseudoplastic shear thinning behavior characteristic of non-Newtonian fluids. Under steady-state shear conditions, the viscosity of the system decreases with increasing shear rate, which is attributed to the macroscopic alignment and disruption of the polymeric network structure induced by shear stress. Dynamic oscillatory testing reveals that the storage modulus (*G′*) exceeds the loss modulus (*G″*) in the high-frequency region, demonstrating an elastic-dominant solid-like behavior that signifies a highly compact and robust internal network formed via electrostatic attractions. Furthermore, creep recovery mechanism analysis indicates that a higher degree of deacetylation of chitosan enhances the resistance against external deformation (lower compliance), demonstrating that the viscoelastic properties can be precisely modulated based on processing conditions to ensure structural stability [137].

In the study of polyelectrolyte coacervates utilizing PSS (4-styrenesulfonate) and PDADMA (poly(diallyldimethylammonium)), a significant theoretical advancement was achieved by unifying the dynamics of viscoelastic liquids across the entire phase diagram. The researchers extended the conventional time temperature (TTS) and time salt (TSS) superposition principles to propose a comprehensive Time Polyelectrolyte Salt Superposition (TPSS) master curve that accounts for simultaneous variations in both actual polyelectrolyte and salt concentrations. These entangled viscoelastic fluids exhibit a self-similar dynamic response, where the viscoelastic spectrum perfectly converges into a single universal curve through the sequential application of a horizontal shift factor for polyelectrolyte concentration and a subsequent shift factor for salt concentration. Specifically, the internal relaxation dynamics of the coacervate follow a highly pronounced power law correlation with changes in actual polymer concentration, providing a versatile framework to quantitatively trace microscopic phase separation interactions from macroscopic rheological data [138].

### 6.2. Wet Interfacial Adhesion

A critical challenge for biomedical coatings is maintaining robust adhesion to tissues or implants submerged in aqueous physiological fluids. Coacervate coatings overcome the hydration barrier by suppressing the interfacial interactions screened by water molecules, utilizing synergistic non-covalent interactions. Inspired by marine organisms, many coacervate systems incorporate catechol-bearing moieties (such as DOPA) or utilize strong electrostatic attractions to displace surface-bound water molecules [93,139].

Coacervate-derived hydrogels address a critical challenge in wet interfacial adhesion by incorporating an active water-repulsion mechanism. This mechanism effectively clears the bound hydration layer on target substrates, enabling direct and robust surface contact.

This system transitions from a fluidic coacervate state to a robust hydrogel network, utilizing synergetic molecular interactions that actively displace water molecules trapped at the tissue interface. By repelling interfacial water, the functional groups within the hydrogel network achieve direct and intimate contact with biological surfaces, establishing strong covalent and non-covalent bonds. This robust underwater bioadhesion provides high interfacial shear strength and structural stability even in dynamic physiological environments, making it highly effective as a tissue sealant and a bioactive patch that promotes accelerated wound healing under wet conditions [125].

The introduction of aromatic residues into polyelectrolyte structures provides a powerful driving force for transforming fluidic coacervates into viscoelastic hydrogels designed for versatile underwater and underoil adhesion. The fundamental mechanism governing this wet interfacial adhesion is rooted in strong cation pi interactions, pi-pi stacking, and hydrophobic behaviors driven by the dense aromatic domains. These robust noncovalent interactions successfully disrupt and penetrate the hydration layer on hydrophilic substrates underwater, while also displacing oil films on oleophilic surfaces underoil.

These aromatic residues dissipate fracture energy through reversible physical crosslinking within the bulk hydrogel. Simultaneously, they establish exceptional interfacial affinity across diverse engineering and biological substrates, thereby expanding the applicability of coacervate-derived adhesives in extreme environments [140].

### 6.3. Elastic Modulus

The elastic modulus (E) of coacervate coatings is highly tunable and uniquely sensitive to environmental stimuli, spanning several orders of magnitude from soft, tissue-mimetic matrices (kPa) to rigid, glassy thin films (MPa to GPa). Unlike conventional thermosetting polymers, the stiffness of a coacervate network is fundamentally governed by its hydration state and charge density. In fully hydrated physiological environments, water molecules act as highly efficient plasticizers that increase the free volume between polymer chains, thereby reducing the net elastic modulus. Conversely, upon controlled dehydration or precise tuning of the ionic strength toward the stoichiometric equivalence (isoelectric) point, the compaction of the electrostatic network significantly restricts segmental chain mobility, inducing a sharp glass transition [141].

The dynamic structural development within hyaluronic acid and chitosan complex coacervates highlights a dramatic evolution of the storage elastic modulus (*G′*) during environmental and compositional gelation transitions. Upon mixing these oppositely charged biopolymers, the linear viscoelastic response is initially dominated by temporary electrostatic physical junctions, which systematically transform into an arrested network exhibiting a sharp rise in elastic modulus. The magnitude and temperature dependency of the plateau elastic modulus are critically determined by the stoichiometric charge ratios, total polymer concentration, and intrinsic chain stiffness. Under optimal pH conditions that maximize ionic pairing, the storage modulus (*G′*) significantly exceeds the loss modulus (*G″*) across a broad frequency spectrum. This dynamic dominant behavior indicates the formation of a robust supramolecular gel architecture with exceptional structural memory and dimensional stability [96].

The manufacturing of polyelectrolyte and clay nanolayered coacervate thin films utilizes an exceptionally high in-plane elastic modulus to deliver superior heat shielding and flame-retardant barrier performance. The dense integration of rigid inorganic clay platelets embedded within the compliant polyelectrolyte coacervate matrix yields a dramatic increase in the Young modulus through a nanoscale composite reinforcement mechanism. This highly elevated elastic modulus prevents structural relaxation, blistering, or thermal cracking under extreme high-temperature exposures. Furthermore, the robust mechanical stiffness provided by the high elastic modulus works cooperatively with the gas barrier properties of the tortuous clay pathway, delaying heat transport and oxygen diffusion to effectively suppress combustion and safeguard underlying substrates [142].

### 6.4. Fatigue Resistance

Coacervate coatings exhibit superior fatigue resistance owing to the inherently thermodynamic and reversible nature of their constituent networks. Under cyclic loading unloading regimes, conventional polymers suffer from accumulated microstructural damage as covalent bonds are irreversibly broken. In contrast, the dynamic linkages within a coacervate matrix can reform during the unloading phase of a cycle through structural hysteresis recovery, effectively healing localized stress concentration before it propagates into catastrophic failure. Concurrently, these networks exploit a hydration lubrication mechanism by maintaining a high-water content of approximately 70 to 80 percent, which drastically lowers the surface friction coefficient. This combination dissipates energy internally through dynamic crosslink rearrangements while minimizing localized frictional heat and mechanical fatigue at the interface. Consequently, these combined mechanisms synergistically suppress coating delamination and fatigue wear [66,143].

The exceptional fatigue resistance of this coacervate-based underwater adhesive is deeply rooted in the highly reversible and dynamic nature of its internal supramolecular network. Unlike conventional structural adhesives that undergo irreversible covalent bond breakage and microstructural damage accumulation under cyclic mechanical stress, this system utilizes noncovalent linkages that can autonomously dissociate and reform. During cyclic loading unloading regimes, these dynamic crosslinks efficiently dissipate macroscopic mechanical energy into heat, preventing stress concentration at the bonded interface. This thermodynamic healing process enables structural hysteresis recovery during the unloading phase. Consequently, the adhesive maintains high bonding strength and structural integrity over multiple cyclic loads without experiencing premature fatigue wear or interfacial delamination [29].

Fatigue resistance is a critical mechanical attribute in the production of monodispersed complex coacervate microcapsules. This durability is essential to withstand severe shear stresses during membrane emulsification and subsequent thermal impacts during spray drying. The core shell or matrix walls formed by complex coacervation exhibit a tough viscoelastic structure that effectively accommodates cyclic physical deformations. The dense yet flexible polyelectrolyte shell dampens external mechanical shocks through continuous chain rearrangements, preventing shell rupture or microcracking under repetitive processing loads. This structural toughness and resistance to mechanical fatigue ensure that the microcapsules retain their architectural stability and core loading efficiency during rigorous industrial manufacturing and long-term storage conditions [144].

In the context of long-term vivo performance, these individual mechanical attributes, including shear thinning behavior, wet interfacial adhesion, elastic modulus, and fatigue resistance, do not operate in isolation. Instead, their synergistic cross-coupling fundamentally determines the structural integrity and operational service life of coacervate coatings under dynamic physiological environments such as cardiovascular pulsation, muscular movement, and joint articulation. Specifically, spatiotemporal synergy operates between shear thinning behavior and fatigue resistance. High shear stresses induced during surgical application or physiological tissue deformation trigger shear thinning and viscoelastic flow. This allows the coacervate fluid to lower its viscosity, rapidly wet substrate micro irregularities, and displace interfacial hydration layers to establish strong initial adhesion. Once the stress relaxes and the coacervate transitions into a gel state, the same reversible noncovalent interactions, such as electrostatic attractions, hydrogen bonding, and cation–π interactions, function as dynamic sacrificial bonds. Under continuous cyclic loading, these bonds dynamically dissociate and reform to dissipate localized strain energy, preventing crack propagation and stress concentration at the interface. Such dynamic crosslinking and interfacial reinforcement mechanisms are increasingly recognized as essential design principles for enhancing the fatigue resistance and long-term mechanical stability of polymeric coatings [145,146]. By combining initial shear-assisted adaptability with ongoing cyclic energy dissipation, this intrinsic mechanical interplay effectively prevents coating delamination and ensures prolonged biofunctionality under continuous tissue deformation.

## 7. Biomedical Applications of Coacervate-Based Biomimetic Coatings

The methodological strategies discussed in the previous section show that coacervate-based coatings can be deposited, stabilized, functionalized, and rendered responsive through multiple design routes. These routes are directly connected to biomedical function because medical interfaces are rarely dry, chemically simple, or mechanically static. Physiological interfaces present distinct chemical and mechanical challenges depending on their clinical context. Wound beds are naturally hydrated and mechanically dynamic, while infected tissues harbor complex biofilms and inflammatory exudates. Furthermore, medical devices are constantly exposed to biofouling agents such as proteins, salts, blood cells, microorganisms, and fluid shear, whereas regenerative interfaces must harmoniously coordinate adhesion, immune responses, degradation, cytocompatibility, and tissue remodeling [147,148,149]. Coacervate-based coatings are particularly suited to these environments because they can form hydrated, deformable, and functionally concentrated interfacial layers under aqueous conditions [150,151]. Rather than acting only as passive barriers, they can behave as adaptive biointerfaces that mediate wet adhesion, therapeutic localization, antibacterial protection, and regenerative signaling.

**Figure 5:**
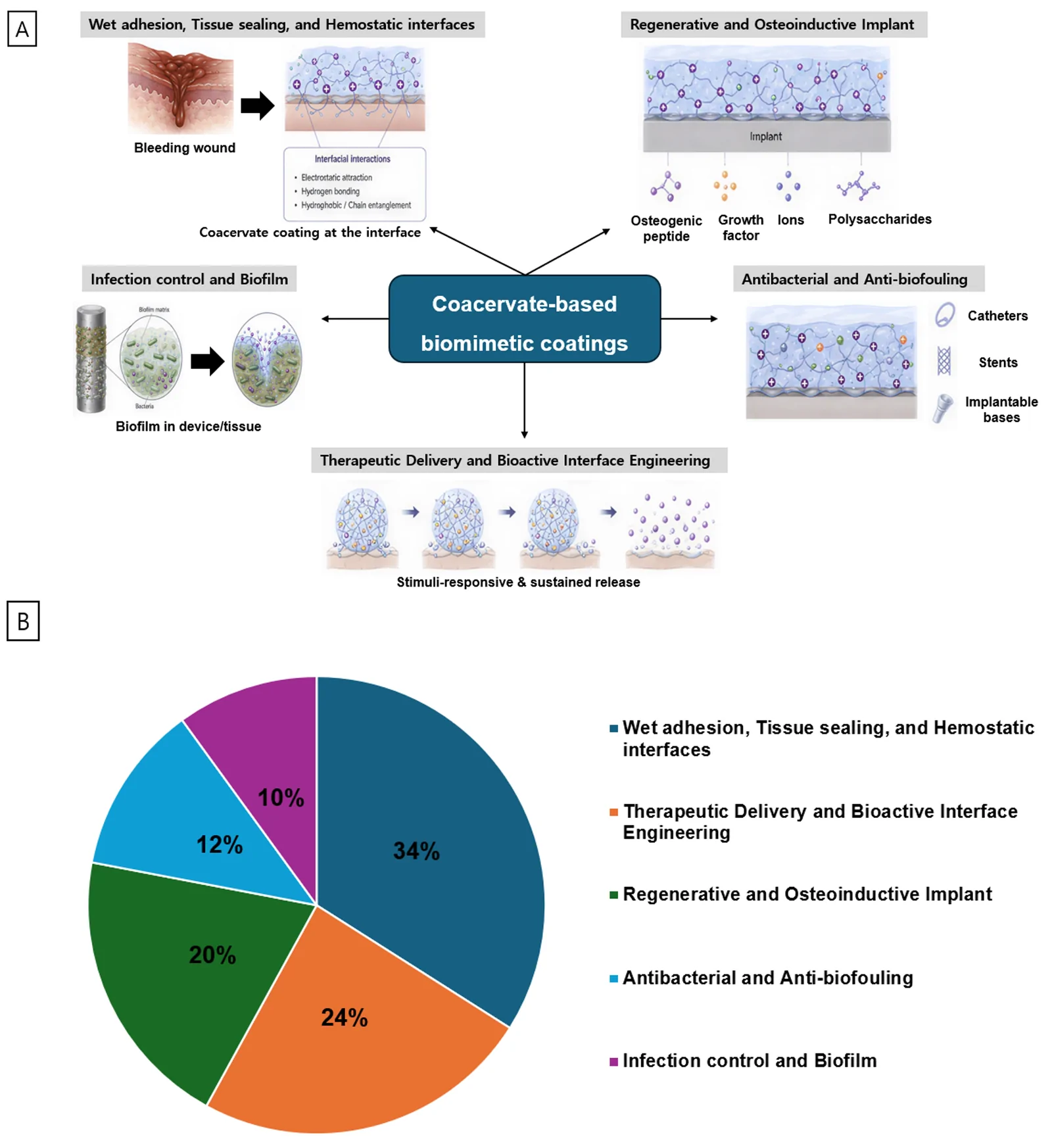
Biomedical application map of coacervate-based biomimetic coatings. (**A**) Coacervate-based coatings can address diverse biomedical interfaces by combining wet adhesion, conformal coverage, cargo retention, environmental responsiveness, and biofunction. (**B**) Percentages in brackets in the module headers and the integrated donut chart represent the relative publication share for each application field, determined through a targeted literature search using the Web of Science Core Collection database (period: 2015–2026).

The major biomedical application areas of coacervate-based biomimetic coatings are summarized in Figure 5. These applications can be broadly grouped into wet tissue adhesion and hemostatic sealing, bone repair and hard-tissue fixation, localized therapeutic delivery and biofilm-associated infection treatment, antibacterial and anti-biofouling device coatings, and regenerative cell–material interfaces [152,153,154,155,156]. Across these areas, the same coating-relevant properties are repeatedly involved, including interfacial wetting, cohesive retention, molecular mobility, cargo partitioning, environmental responsiveness, and biological compatibility.

To provide actionable translational insights, the technologies discussed across these application areas are critically categorized according to their current developmental maturity. While standard commercial products represent clinically established benchmarks, the vast majority of coacervate-based systems currently remain at the preclinical stage (in vivo animal models) or early proof-of-concept phase (in vitro and ex vivo evaluations). Explicitly distinguishing these developmental stages is essential to evaluate whether reported functional performance reflects true translational readiness or fundamental efficacy observed under controlled laboratory conditions.

### 7.1. Wet Adhesion, Tissue Sealing, and Hemostatic Interfaces

Currently, clinically established benchmarks, such as cyanoacrylates, fibrin sealants, albumin-glutaraldehyde glues, and synthetic polyethylene glycol (PEG)-based hydrogels, are widely implemented in surgical practice for tissue sealing, hemostasis, and device fixation [147,148]. However, these commercial standards often suffer from distinct translational drawbacks, including poor wet adhesion, excessive swelling, brittleness, rapid degradation, or local cytotoxicity in hydrated, dynamic physiological environments. To overcome these clinical limitations, coacervate-based biomimetic coatings have been aggressively developed as alternative biointerfaces; however, virtually all coacervate formulations currently exist at either the preclinical stage (for example, in vivo rodent/porcine defect models [152,157]) or the in vitro proof-of-concept stage (for example, recombinant protein design or model substrate testing) [158,159]. While these early-stage coacervates demonstrate exceptional underwater spreading, self-healing, and adaptive cargo delivery, transitioning them from proof-of-concept formulations to clinically approved materials requires addressing key translational bottlenecks, such as long-term shear stability, batch-to-batch scalability, and physiological clearance. 

Wet adhesion is one of the most direct biomedical applications of coacervate-based biomimetic coatings. While conventional medical adhesives like cyanoacrylates, fibrin, albumin, and PEG-based sealants are standard options for tissue sealing and wound closure, they face distinct operational drawbacks. These systems often exhibit limited wet adhesion, inadequate cohesive strength, and unfavorable physical or biological responses, such as excessive swelling, rapid degradation, brittleness, or tissue toxicity [147,148]. These limitations are particularly important because most surgical wounds and traumatic tissue interfaces are hydrated, protein-rich, and mechanically active. Coacervate-based adhesives offer an alternative design logic by forming water-rich condensed phases that can spread over wet tissue while maintaining cohesive integrity [134,160,161].

The adhesive performance of coacervates is closely related to the interfacial principles described in Section 2. A dense coacervate phase can adapt to irregular tissue surfaces, tolerate interfacial water, and establish multiple non-covalent interactions with biological substrates. Bioinspired adhesive systems are particularly instructive in this regard because wet adhesion can arise from hydrogen bonding, hydrophobic interactions, aromatic interactions, metal coordination, chain entanglement, and dynamic covalent chemistry [151,162]. These interactions allow coacervate-based adhesives to behave not only as surface glues, but also as deformable interfacial layers that dissipate stress and maintain contact with moving tissues.

The dynamic phase-separated mussel adhesive protein hydrogel was developed by methacrylating mussel adhesive proteins and then induced phase separation before in situ photopolymerization [152]. In this system, mussel adhesive proteins provided DOPA-rich and charged motifs that promoted molecular condensation through non-covalent interactions, while methacrylation enabled subsequent covalent crosslinking. This design directly reflects the general logic of coacervate-based wet adhesion, whereby a liquid-like condensed phase first improves interfacial adaptation, and later stabilization reinforces the coating after deposition. The resulting hydrogel showed tunable mechanical properties, strong tissue adhesion, self-healing behavior, antibacterial and antioxidant activity, and wound-healing potential. Therefore, this study is useful as a representative case because it demonstrates that coacervation can simultaneously contribute to wet adhesion, mechanical adaptability, and biological function rather than serving only as a precursor-forming step.

Carboxymethyl Poria cocos polysaccharide (CMP)/Tannic Acid (TA)/Fe^3+^ coacervates may also be effective for treating localized tumors [158]. Although the main application of this system was photothermal therapy, it provides an informative wet-adhesive coating model because the coacervate was injectable, fluidic, and capable of spreading over hydrated tissue surfaces. The material was formed through coordination interactions among carboxymethyl Poria cocos polysaccharide, tannic acid, and Fe^3+^, resulting in a condensed phase with good wettability and underwater adhesion. In biological tissue tests, the coacervate adhered to wet tissues such as skin, muscle, liver, and fat, and its tissue retention contributed to sustained local therapeutic effects. This example shows how coacervate-based coatings can exploit wet spreading and interfacial retention to remain localized at irregular biological surfaces.

Protein-engineered coacervate adhesives further illustrate how wet adhesion can be programmed through molecular design. Liang et al. developed an ELP–barnacle cement protein fusion adhesive, E110B, by combining the thermoresponsive phase transition of elastin-like polypeptides with the self-assembling behavior of barnacle cp19k [159]. The fusion protein underwent temperature-dependent phase transition and formed supramolecular nanofibers, and both processes contributed to enhanced adhesive strength. Although this system was mainly evaluated on hard substrates rather than soft tissue interfaces, it holds significant relevance for biomedical coating design. Specifically, it demonstrates that sequence-defined recombinant proteins can successfully integrate phase transition, self-assembly, scalability, and biocompatibility within a single adhesive platform.

A critical comparison of these representative systems reveals that their operational performance and translational potential are strongly governed by underlying formulation differences, specifically in crosslinking chemistry, molecular architecture, and mechanical compliance. First, a fundamental trade-off exists between purely non-covalent dynamic networks and chemically reinforced hybrid systems. Formulations relying strictly on supramolecular coordination and hydrogen bonding (such as CMP/TA/Fe^3+^ [158]) offer immediate fluidic adaptivity and excellent underwater spreading over wet tissues. However, their cohesive integrity remains highly vulnerable to physiological pH, ionic strength, and fluid dilution, which risks premature washout in dynamic environments. In contrast, dual stabilized platforms (such as methacrylated mussel adhesive proteins [152]) overcome this limitation by decoupling interfacial adaptation from cohesive reinforcement: initial liquid state spreading is followed by secondary covalent photocrosslinking. While this secondary crosslinking provides the high shear resistance necessary for tissue sealing, it introduces external operational requirements, such as UV or visible light exposure, during surgical procedures. Second, reported discrepancies in adhesive performance across literature often stem from a mismatch between formulation design and test substrate characteristics. For instance, protein engineered systems featuring thermoresponsive nanofibrillar assembly (such as ELP barnacle fusion E110B [159]) exhibit exceptionally high shear strength on rigid, nondeformable substrates like glass or titanium. However, when translated to dynamic, soft tissue interfaces (such as liver, lung, or heart tissues), such rigid nanofibrillar networks lack the viscoelastic compliance required to dissipate strain during continuous tissue movement, frequently leading to localized stress concentration and delamination. Conversely, fluidic coacervates that excel at soft tissue conformability often fail to withstand high arterial burst pressures unless integrated with rapid in situ solidifying triggers. Therefore, evaluating coacervate adhesives requires critical consideration of whether high reported values reflect true physiological efficacy or are merely artifacts of rigid substrate testing.

For tissue sealing and hemostasis, adhesion alone is not sufficient. The coating should form a continuous barrier, resist leakage, accommodate tissue movement, and remain functional under blood or body fluid exposure. Biomimetic wet-tissue adhesives and hemostatic coacervate-forming systems have been developed to seal wounds, control bleeding, and support wound healing under emergency or non-pressing conditions [134,160,161,163,164]. The representative studies described above suggest that the most important design requirement is a balance between rapid interfacial spreading and post-deposition cohesion. A coating that is too fluid may disperse away from the wound, whereas a coating that hardens too rapidly may fail to conform to tissue deformation.

### 7.2. Bone Repair and Hard-Tissue Fixation

Coacervate-based coatings and adhesives are also being developed for hard-tissue applications, including bone repair, cartilage repair, pulp capping, remineralization, and cranial flap fixation [165]. Compared with soft tissue sealing, hard-tissue interfaces impose additional requirements because the material must adhere to wet mineralized surfaces while supporting mechanical stability, infection control, and osteogenic or mineral-associated activity [92,150,166,167]. In this context, coacervates are attractive because their composition can be tuned to include cationic polymers, phosphate-rich components, mineral-binding motifs, antibacterial groups, and osteogenic cues.

A representative example is the wet-adhesive and antibacterial PAH (Poly (allylamine) hydrochloride)-TPP (Tripolyphosphate) (PAH-TPP) coacervate developed for cranial flap fixation [92]. This system was designed to address several clinical needs at once, including strong fixation, cytocompatibility, antibacterial protection, injectability, shape adaptability, anti-swelling behavior, and self-mineralizing capability. PAH contributes cationic antibacterial activity, whereas TPP can interact with calcium ions and support mineral formation. In this way, the coacervate phase functions not only as an adhesive matrix but also as a multifunctional hard-tissue interface that combines fixation, anti-infection activity, and osteogenic support.

Other hard-tissue-related coacervate systems further expand this design space. Wet-resistant osteogenic nanocomposite adhesives have been explored for controlling bleeding and infection after sternotomy, where a material must resist wet mechanical conditions while supporting healing at a bone-rich surgical site [166]. Calcium polyphosphate coacervate composites have also been investigated for dental pulp capping, suggesting that phosphate-rich coacervate materials can be relevant to mineralized tissue repair beyond orthopedic fixation [167]. In addition, calcium coacervate systems and self-assembling peptide-based approaches have been examined for remineralization of artificial caries lesions, indicating that coacervate-like mineral delivery may be useful in dental hard-tissue regeneration [150].

A critical evaluation of these hard tissues coacervate formulations demonstrates that their functional outcomes and biological performance are strongly influenced by trade-offs between mineralizing kinetics, mechanical load bearing capacity, and local cytocompatibility. First, a primary mechanistic distinction lies in how different formulations balance structural cohesion against osteogenic ionic activity. Systems relying on high densities of polycations or inorganic polyphosphates (such as PAH TPP [92] or calcium polyphosphate coacervates [167]) efficiently recruit calcium ions and stimulate mineral deposition. However, excessive polycationic charge density or rapid polyphosphate dissolution can induce local cytotoxicity and trigger detrimental tissue inflammation. Conversely, incorporating inorganic fillers or nanocomposites (such as in sternotomy adhesives [166]) enhances wet shear strength and dimensional stability, yet overly dense mineral filler loading often restricts polymer chain mobility, thereby hindering interfacial wet spreading and retarding endogenous matrix remodeling. Second, inconsistencies in reported bone regeneration and remineralization efficiencies across literature frequently stem from structural variations in the organic template and local ion release kinetics. For instance, self-assembling peptide coacervates [150] offer precise spatial control over hydroxyapatite nucleation, mimicking native bone extracellular matrix. Nevertheless, these purely supramolecular peptide assemblies often lack sufficient early stage burst pressure resistance and mechanical shear strength required for macroscale orthopedic fixation under physiological load bearing conditions. Therefore, optimizing hard tissue coacervates necessitates careful tuning of inorganic mineral ratios, charge balances, and crosslinking densities to achieve an optimal equilibrium between early mechanical stabilization and long-term tissue integration.

### 7.3. Localized Drug Delivery and Biofilm-Infiltrating Coatings

Another major application of coacervate-based coatings is localized therapeutic delivery. The dense coacervate phase can retain proteins, peptides, nucleic acids, small-molecule drugs, antimicrobial agents, and nanoparticles through electrostatic interactions, hydrogen bonding, hydrophobic association, affinity-based partitioning, or environmentally responsive interactions [129,168,169,170,171,172,173,174]. This allows coacervate coatings to function as local drug reservoirs at tissue, wound, tumor, or device interfaces [129,175,176]. Compared with freely diffusing drug solutions, coacervate phases can prolong local retention, while preserving molecular mobility and environmental responsiveness that are difficult to achieve in highly crosslinked matrices. Coacervate-mediated delivery has been explored across diverse therapeutic contexts. However, its primary significance lies not merely in cargo diversity, but in the ability of the condensed phase to preserve and optimize the handling of fragile or functionally complex biomolecules.

For protein delivery, an mPEGylated coacervate was used to load Aleuria Aurantia lectin (AAL), protecting the anticancer lectin and enabling pH-responsive release under mildly acidic tumor-like conditions, which helped sustain AAL exposure and suppress recurrence of pancreatic cancer cell proliferation [169]. Redox-responsive peptide coacervates further extended this strategy by sequestering tissue plasminogen activator (tPA) within NADPH/arginine-rich peptide droplets, adding RGD-mediated thrombus targeting, and triggering tPA release through redox-induced coacervate dissolution [129]. In intracellular delivery, phase-separating peptide coacervates demonstrate that sequence-level modifications in histidine-rich peptides can finely tune droplet viscoelasticity, membrane interactions, cargo uptake, and release kinetics. This precise physical control enables the effective delivery of diverse macromolecules, including proteins, nucleic acids, and CRISPR/Cas9 components [171]. For RNA therapy, SHMP/SS-31 coacervate artificial cells loaded with miRNA-223, and further protected by erythrocyte membrane coating, improved miRNA stability and cytoplasmic delivery while combining macrophage repolarization with antioxidant effects in acute lung injury [172]. More recently, nucleic acid coacervates wrapped in a peptide-polyphenol network were engineered as drug-free protocells targeting breast cancer cells. Entering cells via dual endocytic pathways, these systems induce mitochondrial dysfunction, endoplasmic reticulum (ER) stress, apoptosis, and pyroptosis without requiring conventional chemotherapeutic agents [168]. Together, these examples show that coacervates are not merely passive carriers for different cargos. Instead, they can function as hydrated therapeutic compartments that integrate cargo enrichment, protection, targeting, responsiveness, intracellular transport, and even direct regulation of cell fate.

A critical evaluation of these localized delivery systems demonstrates that reported inconsistencies in drug release durations and intracellular delivery efficiencies are strongly dictated by underlying formulation variables, such as charge density, host guest affinity, and environmental responsiveness mechanisms. First, a major discrepancy across literature concerns the release kinetics of macromolecular cargos from coacervate carriers, ranging from rapid burst release within hours to sustained release over several days. Formulations relying purely on weak electrostatic partitioning (such as simple polycation polyanion droplets) often suffer from premature cargo leakage due to competitive displacement by physiological ions and proteins. In contrast, incorporating secondary affinity interactions or stimulus responsive hydrophobic components (such as mPEGylated lectin complexation [169] or redox sensitive peptide assembly [129]) stabilizes the condensed phase, effectively extending therapeutic exposure. However, overly tight cargo partitioning or high crosslinking density within the coacervate matrix can impede molecular mobility, resulting in incomplete drug release and reduced bioavailability. Second, for nucleic acid and intracellular therapeutic delivery, differences in cellular uptake pathways and endosomal escape efficiency stem from subtle sequence level or structural modifications in the coacervate template. For instance, histidine rich peptide coacervates [171] achieve effective cytosolic release by leveraging pH sensitive membrane destabilization. Conversely, systems relying strictly on dense surface coatings (such as membrane wrapped nucleic acid protocells [168]) prioritize targeted cellular internalization but may exhibit variable cytoplasmic bioavailability depending on whether they escape endosomal degradation or undergo lysosomal routing. Therefore, rational design of delivery coacervates requires balancing thermodynamic cargo retention against kinetic release responsiveness under physiological conditions.

Biofilm-associated infections represent a particularly challenging target for localized delivery because the extracellular polymeric matrix limits antimicrobial penetration, alters local pH and oxygen levels, protects dormant or slow-growing bacteria, and promotes treatment recurrence [162,177]. Liposome-based anti-biofilm strategies effectively overcome conventional treatment barriers by enhancing local antibiotic availability, biofilm penetration, and antimicrobial retention. These improvements have been successfully demonstrated in advanced formulations, including cationic or PEG-modified liposomes, lysozyme-associated gentamicin liposomes, and antimicrobial peptide/antibiotic-loaded systems. However, a more directly relevant coacervate-based example is the pH-responsive alginate–caseinate nano-coacervate system developed for oral biofilm-associated infections [178]. Sodium alginate and sodium caseinate were assembled into mucoadhesive nano-coacervates under acidic conditions and used to encapsulate nisin, a natural antimicrobial peptide. The system showed higher nisin encapsulation at pH 5 than at neutral pH, sustained nisin release in simulated salivary fluid, and stronger inhibition of multidrug-resistant oral biofilm-forming bacteria, including *Enterococcus faecalis*, and *Staphylococcus epidermidis*, than free nisin. Importantly, the nisin-loaded nano-coacervates inhibited both biofilm formation and preformed biofilms, suggesting that coacervate carriers can improve local antimicrobial persistence and biofilm control in the oral cavity.

This application is mechanistically consistent with the phase-separated nature of biofilm matrices. Gupta and Guptasarma showed that the *E. coli* nucleoid-associated protein HU can coacervate with DNA and lipopolysaccharide to form a biofilm-simulating extracellular polymeric substance, and that lipopolysaccharide (LPS) modulates the liquidity of HU–DNA condensates while allowing *E. coli* cells to adhere to or advance into the condensate [179]. This finding suggests that biofilm extracellular polymeric substance (EPS) is not merely a passive diffusion barrier, but a hydrated and dynamically organized condensed environment. Consequently, coacervate-based coatings and carriers are uniquely suited for biofilm-targeted therapies. Their soft, hydrated, and interracially mobile condensed phases can be tailored to interact with charged EPS networks, thereby enhancing local retention and providing sustained antimicrobial release within biofilm matrices.

A critical analysis of biofilm infiltrating coatings reveals that variations in reported anti biofilm efficacy stem primarily from structural and electrostatic mismatches between the coacervate vehicle and the extracellular polymeric substance matrix. First, a persistent challenge in treating mature biofilms is the trade-off between vehicle stability and matrix penetration. Formulations possessing high cationic charge densities exhibit strong mucoadhesion and affinity for negatively charged bacterial membranes. However, such strongly charged coacervates often become electrostatically trapped at the outer periphery of the extracellular polymeric substance matrix, hindering deep spatial penetration into dormant bacterial niches. Conversely, nano coacervates engineered with pH responsive charge conversion or dynamic interfacial mobility (such as alginate caseinate systems [178] adapt their surface potential to the acidic microenvironment of oral or wound biofilms. This local adaptability facilitates deeper matrix infiltration and sustained antimicrobial release, although their physical stability remains highly sensitive to localized pH fluctuations. Second, discrepancies between in vitro biofilm inhibition assays and in vivo therapeutic outcomes frequently highlight the dynamic physical nature of native biofilms. As demonstrated by biomimetic nucleoid condensates [179], the extracellular polymeric substance is an actively phase separating, viscoelastically evolving network rather than a static barrier. Consequently, passive coacervate carriers that lack active-matrix degrading enzymes or dynamic liquid-like properties often fail to disrupt preformed, dense biofilms under physiological fluid shear. Evaluating anti biofilm coacervate coatings thus requires assessing not only early phase bacterial growth inhibition, but also long-term matrix destabilization and viscoelastic compliance within hydrated infection sites.

### 7.4. Antibacterial and Anti-Biofouling Medical Device Coatings

Medical devices such as catheters, stents, tubes, and implant surfaces are highly susceptible to protein adsorption, bacterial adhesion, biofilm formation, thrombosis, and mineral deposition. These complications arise because device surfaces are continuously exposed to biological fluids, salts, proteins, cells, microorganisms, and mechanical flow [180]. Coacervate-based and coacervate-inspired coatings offer a promising route to address these problems by combining hydration, lubricity, anti-adhesion, antibacterial activity, cargo retention, and long-term interfacial stability [127,181,182,183].

Interface engineering offers another layer of control for coacervate-based coating design. Yin et al. showed that coacervate microdroplets can be coated with surfactant-mediated membrane-like layers through electrostatic complexation between charged surfactants and polyelectrolytes [127]. This interfacial layer improved droplet stability against coalescence and pH- or salt-induced disassembly, while also tuning permeability and molecular partitioning. Although this study was performed in model coacervate microdroplets rather than on implanted devices, it is highly relevant to medical coatings because it demonstrates how the boundary of a condensed phase can be engineered independently from its interior. For device applications, this suggests that coacervate-inspired coatings could be designed with a hydrated bulk phase for cargo retention or lubrication and a regulated interfacial layer for durability, selective transport, and anti-biofouling performance.

The broader usefulness of polyelectrolyte–surfactant complexes (PESCs) come from their tunable internal organization. Unlike simple polymer coatings, these complexes combine electrostatic association with surfactant-derived hydrophobic domains. This dual nature allows them to accommodate molecules of varying polarity and hydrophobicity, while simultaneously tuning their phase behavior and rheological properties [182].

Their structure can be regulated by the polyelectrolyte architecture, surfactant type, charge ratio, salt concentration, concentration pathway, and the presence of stabilizing hydrophilic segments. This tunability is important for medical coatings because antibacterial or anti-biofouling function often requires more than surface immobilization. The coating must remain processable during deposition, stable after contact with biological fluids, and capable of balancing hydration, cargo retention, and interfacial interaction. In this sense, PESCs provide a formulation logic for designing coatings whose assembly, solubility, stability, and biological function can be coordinated within one material system.

Catheter coatings provide a representative example of this design strategy. Yu et al. developed a regulable polyelectrolyte–surfactant complex coating for antibacterial biomedical catheters by assembling an organosilicon quaternary ammonium surfactant with an adjustable polyelectrolyte containing zwitterionic, crosslinkable, and anionic segments [183]. Specifically, each functional component was designated for a distinct surface mechanism: the quaternary ammonium surfactant imparted non-leaching contact-killing activity, whereas the zwitterionic sulfobetaine methacrylate (SBMA) moieties formed a hydrated anti-biofouling interface. Furthermore, the anionic groups facilitated complexation with the cationic surfactant, and the hydroxyl/siloxane chemistry enabled subsequent post-coating crosslinking. Because the complex was soluble in alcohol, it could be rapidly deposited onto catheters and other irregular medical devices by dip-coating, while thermal curing converted the adsorbed complex into a more durable interfacial network. As a result, the coated catheter reduced bacterial adhesion, protein adsorption, blood cell attachment, and friction, while maintaining antibacterial performance under long-term immersion, high-salt, organic-solvent, and flow-related conditions.

Together, these studies indicate that antibacterial and anti-biofouling medical device coatings do not need to rely only on leachable antibiotics or short-lived antimicrobial release. Nonleaching contact-killing motifs, hydration-mediated anti-adhesion, low-friction interfaces, tunable complexation, and engineered interfacial stability can be integrated into coacervate-inspired coating systems to suppress bacterial colonization while improving long-term device compatibility.

A critical evaluation of these medical device coating formulations highlights that reported variations in long-term antibacterial efficacy and biofouling resistance stem from underlying formulation trade-off, particularly regarding mechanical durability under fluid shear, surface inactivation by dead microbial debris, and phase behavior stability. First, a major mechanistic distinction exists between nonleaching contact killing systems and active release coacervate coatings. Formulations relying purely on cationic contact killing motifs (such as quaternary ammonium PESC complexes [183] avoid premature drug depletion and systemic toxicity risks associated with leachable antibiotics. However, a well-known limitation of purely contact active coatings is the accumulation of dead bacterial debris and adsorbed proteins over time, which masks the underlying antimicrobial groups and gradually diminishes contact killing performance. To overcome this fouling-induced deactivation, incorporating zwitterionic segments (such as SBMA [183] or surfactant regulated interfacial shells [127] is necessary to form a dense hydration barrier that repels dead cells. Yet, increasing the fraction of highly hydrophilic zwitterionic moieties can weaken internal hydrophobic coacervation, making the coating more susceptible to swelling or detachment under dynamic physiological fluid flow. Second, discrepancies between short-term in vitro antimicrobial assays and long-term in vivo medical device performance frequently arise from differences in crosslinking density and deposition processing. While fluidic or weakly crosslinked polyelectrolyte surfactant complexes provide convenient solvent dip coating and excellent initial lubrication, they often undergo slow dissolution or structural reorganization upon exposure to high ionic strength blood or urinary fluids. Post deposition curing or covalent stabilization significantly enhances interfacial durability against mechanical shear, but excessive crosslinking alters the hydration capacity and chain mobility of the coacervate matrix, potentially compromising its anti-biofouling properties. Therefore, designing durable medical device coatings requires a precise formulation balance that maintains surface hydration and active antimicrobial exposure without sacrificing physical stability under continuous fluid flow.

### 7.5. Regenerative Biointerfaces and Cell–Material Interaction

In tissue engineering and regenerative medicine, the surface of an implant, scaffold, or wound-contacting material controls the earliest biological events after implantation. Protein adsorption, cell attachment, macrophage response, vascularization, matrix deposition, and fibrous encapsulation are all influenced by the local cell–material interface [149]. Coacervate-based coatings can regulate these processes because they can maintain hydration, present soft ECM-like environments, localize bioactive molecules, and respond to local biological cues [155,171,172,174]. These features are particularly advantageous in pathological regenerative microenvironments. In these tissue sites, healing is constrained not simply by insufficient bioactivity, but by a complex interplay of infection, oxidative stress, severe inflammation, metabolic imbalance, and poor vascularization.

Beyond molecular delivery, coacervates can also function as cell-supportive transport matrices. Thermoresponsive complex coacervates formed from poly(N-isopropylacrylamide) (PNIPAAm)-grafted chitosan (CHT) and hyaluronic acid (HA) were developed as injectable carriers for cell-laden liquid-core (LC) capsules [174]. The material showed shear-thinning behavior for injection and underwent rapid sol–gel transition at physiological temperature, enabling in situ retention after delivery. Importantly, the system supported high viability of human adipose stem cells and allowed hierarchical organization by separating the cell-laden LC capsules from the surrounding coacervate transport matrix. This example extends the role of coacervates from bioactive coatings or drug reservoirs to regenerative carriers that can localize and organize living therapeutic components at the target site.

Recent examples show how coacervate-based systems can be adapted to regenerative microenvironments by coupling local biological cues with therapeutic function. In diabetic wound healing, glucose-responsive coacervate protocells were generated from diethylaminoethyl-dextran/dsDNA microdroplets and coated with a phospholipid membrane to improve biocompatibility [157]. Glucose oxidase (GOx) and copper peroxide nanodots (CuNDs) were incorporated into the coacervate droplets, allowing the hyperglycemic wound environment to trigger a cascade reaction that produced antibacterial ROS. In diabetic mouse wounds, this design promoted antibacterial activity and accelerated wound closure. This study is useful as a regenerative interface example because the coacervate did not simply act as a passive reservoir. Instead, it converted a disease-associated cue, glucose, into a local therapeutic response.

Coacervate-based strategies have also been applied to scaffold functionalization under diabetic regenerative conditions. For diabetic heart valve regeneration, peptide coacervate–Prussian blue hybrid supraparticles were incorporated into decellularized scaffolds to regulate oxidative stress and improve endothelialization [184]. In this system, the peptide coacervate was formed from oppositely charged mitochondria-targeted antioxidant peptide SS-31 and REDV-derived endothelial cell-adhesive peptides, while Prussian blue nanoparticles (PBNPs) provided enzyme-mimetic ROS-scavenging activity. Each component addressed a specific barrier in the diabetic repair microenvironment: SS-31 protected mitochondria, the REDV motif promoted endothelial cell adhesion, and PBNPs reduced oxidative stress. The resulting scaffold decreased endothelial cell apoptosis, supported endothelial proliferation and migration, suppressed inflammatory macrophage responses, and promoted endothelial layer formation and ECM remodeling in diabetic rabbit vascular models. This example shows how coacervate-based interfacial modification can be used to tailor scaffolds for disease-impaired regeneration rather than for generic tissue repair.

A critical comparison of these regenerative biointerfaces demonstrates that reported inconsistencies in tissue integration and cell retention efficiency stem from underlying formulation differences, particularly regarding the trade-off between viscoelastic softness and structural stability, as well as the balance between stimulus triggered activity and long-term extracellular matrix remodeling. First, a fundamental challenge in cell and therapeutic delivery using coacervate matrices is balancing injectability against long-term physical matrix integrity. Formulations optimized for cell encapsulation or shear thinning injection (such as thermoresponsive PNIPAAm grafted chitosan coacervates [174]) provide excellent cytocompatibility, hydrated cell supportive niches, and rapid shape adaptation. However, these highly hydrated, physically crosslinked networks often possess low storage moduli and rapid degradation kinetics under dynamic tissue deformation, which can lead to premature matrix dissolution before endogenous extracellular matrix deposition occurs. Conversely, introducing dense chemical crosslinking or rigid mineral nanocomposites to increase mechanical durability can impair spatial cell migration, compromise nutrient diffusion, and trigger localized foreign body encapsulation. Second, for pathological regenerative microenvironments such as diabetic wounds or ischemic tissue, discrepancies between initial microenvironment regulation and sustained tissue repair arise from variations in cue responsiveness and temporal stability. Smart coacervates engineered with disease responsive cascades (such as GOx CuND protocells [157] or ROS scavenging peptide supraparticles [184] excel at mitigating acute physiological barriers, such as localized oxidative stress or hyperglycemic bacterial growth. Nevertheless, a well-recognized limitation of these bioresponsive systems is their transient operational lifespan. Once the initial therapeutic trigger or bioactive peptide is consumed, the residual coacervate scaffold must seamlessly support long-term cell infiltration, endothelialization, and vascularization. Achieving a synchronized transition from early biochemical microenvironment modulation to long-term biological tissue integration remains a key formulation bottleneck that dictates true clinical translation.

## 8. Current Challenges and Future Perspectives

Despite the considerable potential of coacervate-based coatings for biomedical interface engineering, several critical challenges must be addressed before their broader translation into practical biomedical and industrial applications [52,53,185,186,187,188,189]. Driven by unique fluid-to-solid phase transitions and non-covalent network formulations, these biomimetic systems offer unprecedented advantages in conformal tissue adaptation and wet-interfacial adhesion. However, moving from controlled, bench-top synthesis to complex in vivo environments and industrial-scale production introduces multifaceted bottlenecks.

A critical barrier to the clinical translation of coacervate-based coating systems lies in their limited long-term stability under physiologically relevant conditions. As highly hydrated, metastable condensed phases governed predominantly by weak non-covalent interactions, coacervates are inherently vulnerable to structural deterioration, including swelling, erosion, dilution-induced dissociation, and loss of mechanical integrity. These limitations may be exacerbated in vivo by a multitude of biological factors, such as enzymatic activity, dynamic ionic environments, protein-mediated interactions, inflammatory processes, and continuous mechanical loading [40,190].

Beyond fundamental material challenges, the scalable manufacturing of coacervate-based coatings remains a significant translational hurdle. The formation and physicochemical properties of coacervates are governed by a complex interplay of environmental and compositional variables, including pH, ionic strength, macromolecular concentration, molecular architecture, mixing sequence, and processing history. As a result, even subtle deviations in formulation or fabrication parameters can lead to pronounced changes in phase behavior, viscoelastic properties, coating uniformity, cargo retention, and adhesive performance [37,70,191,192]. Although this sensitivity enables precise control over material characteristics in research settings, it poses considerable obstacles to process standardization, large-scale production, and batch reproducibility.

A further priority for the field is the rigorous evaluation of coacervate-based coatings in physiologically relevant in vivo environments. Although substantial progress has been achieved through in vitro studies, the biological performance of coacervate coatings remains insufficiently understood under the complex and dynamic conditions encountered in living systems. Upon implantation, these materials must function in the presence of enzymatic degradation pathways, inflammatory and immune responses, tissue remodeling processes, interstitial and vascular fluid dynamics, and persistent mechanical stresses. The interplay among these factors may profoundly influence coating stability, degradation pathways, bioactive cargo release, and therapeutic outcomes [193,194].

To overcome these interconnected barriers, future research must shift from empirical formulation toward molecularly engineered stabilization and stimuli-responsive crosslinking strategies. To mitigate premature dissociation and creep without sacrificing the fluid-like wetting advantages of coacervates, subsequent efforts should focus on integrating orthogonal, bioorthogonal, or reversible covalent chemistries (such as click chemistry, photo-initiated radical polymerization, or dynamic covalent bonding). This allows the coacervate to seamlessly spread and adhere to wet biological substrates as a fluid, followed by an on-demand “locking” mechanism to secure long-term mechanical resilience. Additionally, introducing supramolecular protective motifs, such as hydrophobic domains or peptide-based self-assembling segments within the polyelectrolyte backbone, can shield the internal electrostatic complexes from high-salt dissociation and enzymatic access, thereby providing a programmable degradation profile tailored to specific tissue-healing timelines [41,195,196,197].

Concurrently, the transition from laboratory-scale fabrication to clinical reality demands the establishment of advanced processing frameworks and predictive, high-fidelity screening platforms [171,198,199,200]. The manufacturing bottleneck should be addressed by adopting automated microfluidic mixing systems and continuous-flow deposition technologies, which provide precise kinetic control over phase separation and eliminate the mixing variations inherent to batch processing. In tandem with hardware scaling, future investigations must replace oversimplified vitro assays with organ-on-a-chip models that incorporate physiological fluid shear, multilineage cell co-cultures, and immune components. By pairing these biomimetic platforms with real-time process analytics and high-throughput screening, researchers can systematically map out quantitative structure-property-performance relationships. This dual-pronged approach will transform coacervate coatings from sensitive laboratory phenomena into highly reproducible, clinically viable therapeutic countermeasures.

The continued expansion of the coacervate materials toolbox will be essential for overcoming current limitations and broadening the functional capabilities of coacervate-based coating systems. In particular, marine-derived biomaterials represent a highly promising source of inspiration for the development of next-generation coacervates, as marine organisms have evolved sophisticated molecular strategies for underwater adhesion, interfacial condensation, self-assembly, and environmental resilience. Advances in marine biotechnology, bioinformatics-guided biomaterial discovery, and biomimetic materials engineering are expected to accelerate the identification of novel polysaccharides, proteins, peptides, and organic–inorganic hybrid components capable of imparting enhanced coacervation behavior, robust interfacial adhesion, improved biological performance, and programmable degradation profiles [201,202,203,204,205]. At the same time, the intrinsic compositional heterogeneity associated with natural-source materials remains a significant challenge. Accordingly, future efforts should emphasize the establishment of standardized extraction, purification, and characterization methodologies to ensure material consistency, reproducibility, and long-term biomedical safety.

Ultimately, accelerating the clinical translation of coacervate platforms will pivot on integrating Artificial Intelligence (AI) and Machine Learning (ML) to navigate the hyper-dimensional parameter space of LLPS [206,207]. Recent advances have demonstrated the feasibility of this digital transformation; for instance, active ML algorithms have been successfully deployed to automatically map condensate phase behaviors [206], while integrated molecular dynamics and ML frameworks now allow mechanistic predictions of protein encapsulation and release dynamics within coacervates [207]. Building on these data-driven foundations, future paradigms will increasingly leverage AI guided generative models to predict coacervation windows and viscoelastic properties of novel polyelectrolyte combinations prior to synthesis, bypassing traditional trial and error approaches. By leveraging data-driven models and algorithmic optimization strategies, these intelligent frameworks can optimize smart nanocarriers to adapt to specific pathological microenvironments [208], refine lipid nanoparticle formulations for complex nucleic acid delivery [209], and design stimuli-responsive nanoplatforms capable of precise release against resistant pathogens [210]. Transitioning from empirical screening to an AI-driven predictive design framework will thus establish a robust paradigm for next generation biomaterial engineering.

In biomedical and eco-friendly applications, evaluating the biodegradation cycles and recyclability of coacervate systems is essential for ensuring long-term environmental sustainability and biocompatibility. Complex coacervates, characterized by reversible phase separation and dynamic non-covalent interactions, offer promising pathways for eco-friendly waste management, including soft disassembly and non-destructive recovery of constituent polymers, aligned with recent supramolecular design strategies for sustainable plastics [211]. Furthermore, the biodegradation kinetics of these coacervate networks can be precisely tailored via enzymatic hydrolysis and microenvironmental pH modulation, sharing structural logic with state-of-the-art biodegradable biocomposites [212]. As recent advances in functional polymer networks and hybrid matrices continuously emphasize the necessity of sustainable material design and structural management [146], incorporating controllable degradation profiles and recyclable mechanisms into coacervate architectures significantly broadens their applicability as next-generation green biomedical materials.

## 9. Conclusions

This review has comprehensively delineated the recent breakthroughs in biomimetic coacervate coatings, tracing their trajectory from the fundamental physicochemical tenets of liquid–liquid phase separation (LLPS) to biomedical application. By elegantly emulating the evolutionary design logic of natural underwater adhesive systems, coacervate-based formulations have emerged as a highly adaptable platform that successfully circumvents the classic constraints of conventional surface treatments. This is achieved particularly through their low interfacial tension, superb conformal wetting, and intrinsically benign, aqueous processing. As systematically evaluated, the strategic integration of marine-derived polysaccharides, sequence-defined recombinant polypeptides, and inorganic reinforcing agents such as polyoxometalates and calcium phosphates has expanded the functionality of these coatings, enabling major advancements in wet tissue sealing, self-mineralizing hard-tissue repair, targeted biofilm infiltration, and the spatiotemporal modulation of disease-impaired regenerative microenvironments.

Looking ahead, translating these sensitive laboratory phenomena into clinically viable, commercial-scale therapeutic countermeasures demands a concerted shift toward standardized, actionable manufacturing paradigms. Given the structural and mechanistic diversity of coacervate systems, defining a single, universal test protocol for industrial batch quality control is practically unfeasible. Instead, future effort should focus on establishing an adaptable quality control framework based on system-specific analytical benchmarks. Specifically, industrial scaling via continuous-flow technologies requires (1) in-line physical monitoring (e.g., dynamic light scattering or turbidity tracking) to maintain droplet size uniformity and phase boundary consistency; (2) standardized rheological profiling to precisely control viscoelastic limits and conformal coverage; and (3) substrate-tailored mechanical benchmarks, such as modified ASTM wet-adhesion tests under simulated physiological environments. Overcoming the intrinsic vulnerability of metastable condensates to in vivo erosion will also require bioorthogonal locking chemistries and supramolecular protective motifs to program degradation timelines. Concurrently, migrating from empirical trial-and-error formulations to predictive manufacturing paradigms will be accelerated by integrating automated microfluidic frameworks with artificial intelligence (AI) or machine learning (ML) architectures optimized for multi-dimensional LLPS parameter spaces. Ultimately, this structured convergence combining standardized analytical testing with predictive engineering will establish biomimetic coacervate coatings as a vital, highly reproducible cornerstone technology for next-generation smart medical devices, implantology, and personalized regenerative medicine.

## Figures and Tables

**Table 1:** Comparative summary of major biomaterial classes for coacervate-based coatings.

Biomaterial Class	Representative Materials	Cost & Scalability	Biocompatibility	Coating Uniformity & Processability	Mechanical Limitations	Reference
Marine-Derived Biopolymers	Alginate, Chitosan, Marine Collagen, Gelatin	Low cost; abundant source with excellent large-scale scalability.	High natural biocompatibility and low immunogenicity; intrinsically biomimetic.	Moderate-to-high; highly sensitive to pH, ionic strength, and batch-to-batch structural variance.	Low cohesion; highly susceptible to ambient hydration and enzymatic degradation without secondary crosslinking.	[94,95,96]
Synthetic & Recombinant Polypeptides	Elastin-like polypeptides (ELPs), Recombinant Mussel Adhesive Proteins (rMAPs)	High cost; complex recombinant expression or chemical synthesis; limited scalability.	High to variable; sequence precision minimizes toxic residues, though potential immunogenic responses depend on non-natural motifs.	High; precise sequence-defined phase behavior enables smooth, highly reproducible nano/micro-layer deposition.	Moderate; flexible and viscoelastic, but lacks extreme shear/compressive strength unless chemically crosslinked.	[99,100,101,102]
Hybrid & Inorganic-Reinforced Coacervates	Silica hybrids, POM-peptide complexes, calcium phosphate/TPP coacervates	Moderate; requires multi-step co-assembly or inorganic nanocluster synthesis.	Moderate-to-high; highly dependent on inorganic dose/leaching (e.g., cytotoxicity of unreacted POMs or heavy ions).	Variable; inorganic phase aggregation may cause local interfacial heterogeneity or increased surface roughness.	High toughness & rigidity; superior load-bearing capacity, but prone to brittleness if inorganic loading is excessive.	[60,91,92,103,104]

**Table 2:** Comparative analysis of complex coacervate-based surface coating strategies, highlighting quantitative metrics for film thickness, batch throughput, and manufacturing costs.

Coating Strategy	Representative Thickness Range	Batch Capacity/Throughput	Relative Mfg. Cost
Direct Deposition (Spin)	10 nm–5 µm	Low (<60 units/h, single wafer)	Moderate
Direct Deposition (Dip)	50 nm–50 µm	High (roll-to-roll/multi-substrate compatible)	Low–Moderate
Direct Deposition (Spray)	100 nm–100 µm	Very high (continuous large-area)	Low
In Situ Coacervation	1 µm–several mm	Moderate (multi-barrel syringe/nozzle-driven)	Moderate
LbL Assembly	1 nm–1 µm (bilayer proportional)	Low (Dip-LbL) to Moderate (Spray-LbL)	High

**Table 3:** Comparative Analysis of Complex Coacervate-Based Surface Coating Strategies for biomedical applications.

Coating Strategy	Strategic Advantages	Primary Disadvantages	Scalability & Manufacturing Challenges	Long-Term Stability	Clinical Translation Potential & Regulatory Barriers	Reference
Direct Deposition of Preformed Coacervates	- Preoptimization & full characterization of bulk rheology prior to application - Simple, rapid processing with fewer steps	- High viscosity limits uniform spreading - Shear sensitivity during handling - Potential poor initial retention on wet substrates	- Scalability: High (compatible with industrial dip/spray/spin processes) - Challenges: Batch-to-batch viscosity variations, handling of condensed viscous phases	- Moderate to Low without secondary crosslinking - Susceptible to erosion under physiological fluid shear	- High for external medical devices/wound dressings - Low risk due to ex vivo formulation control	[106,107]
In Situ Coacervation at Target Interface	- Conformal contact on irregular, curved, or wet tissue interfaces - Minimally invasive delivery (injectable/sprayable)	- Precise spatiotemporal control required - Risk of premature gelation or washout/dilution before consolidation	- Scalability: Moderate - Challenges: Complex multi-barrel packaging/delivery systems, sensitive trigger kinetics under dynamic biological fluids	- Moderate - Dependent on local environmental trigger stability and rapid phase conversion	- High for tissue sealants, homeostatic agents, and surgical glues - Requires strict biocompatibility validation for in situ triggers	[114,115,116]
Layer-by-Layer (LbL) & Hybrid Multilayers	- Nanoscale precision over thickness and architecture - High cargo capacity for multifunctional/sequential release	- Time-consuming, labor-intensive assembly - Low thickness buildup per cycle	- Scalability: Low to Moderate - Challenges: Slow diffusion-limited assembly; requires advanced automation (e.g., fluidics/spray-assisted LbL)	- High due to dense interchain ionic crosslinking networks	- Moderate for localized medical implants/stents - High manufacturing costs and long quality control processes pose hurdles	[121,122]
Post-Coating Stabilization	- Exceptional mechanical durability and resistance to physiological shear/washout - Prevents burst release/leaching of therapeutics	- Reduced matrix flexibility/softness - Potential loss of bioactivity/responsiveness due to over-crosslinking	- Scalability: High - Challenges: Precise control of secondary trigger conditions (UV light, chemical reagents, ionic bath changes)	- Very High - Robust against ambient environmental fluctuations (pH, hydration, ionic strength)	- Moderate to High - Residual crosslinking reagents/initiator toxicity must be strictly evaluated by regulatory bodies	[123,124,125,127]
Stimuli-Responsive Coatings	- Dynamic adaptability to microenvironmental triggers (pH, ROS, enzymes) - Smart, on-demand drug release profile	- Complex synthetic design - Narrow therapeutic window or triggering thresholds in dynamic biological systems	- Scalability: Low to Moderate - Challenges: Multistep polymer synthesis, batch consistency, precise responsiveness tuning	- Tunable (designed for controlled degradation or prolonged stability until triggered)	- Moderate - High efficacy for targeted therapies, but faces stringent approval pathways due to complex degradation pathways	[37,128,129,131]

## Data Availability

No new data were created or analyzed in this study. Data sharing is not applicable to this article.
